# Combined associations of the frailty index and CHG index with cardiometabolic multimorbidity: a longitudinal study from the CHARLS cohort

**DOI:** 10.3389/fnut.2026.1867031

**Published:** 2026-06-05

**Authors:** Lin Huang, Xuefang Yan, Youqin Wang, Yun Ti, Peili Bu, Jingyuan Li

**Affiliations:** State Key Laboratory for Innovation and Transformation of Luobing Theory, Key Laboratory of Cardiovascular Remodeling and Function Research of MOE, NHC, CAMS and Shandong Province, Department of Cardiology, Qilu Hospital of Shandong University, Jinan, China

**Keywords:** cardiometabolic multimorbidity, cholesterol, high-density lipoprotein, and glucose index, dynamic change, frailty index, population-based cohort

## Abstract

**Introduction:**

Frailty and metabolic dysfunction are major contributors to cardiometabolic multimorbidity (CMM). However, the joint influence of frailty and insulin resistance on the onset of CMM is still insufficiently understood. This research aimed to examine the association between FI-CHG [a novel integrative metric combining the frailty index (FI) and the cholesterol-high-density lipoprotein-glucose (CHG) index] and incident CMM.

**Method:**

Overall, 8,543 participants aged ≥45 years without CMM at baseline from the China Health and Retirement Longitudinal Study (CHARLS) were recruited. Multivariable Cox regression models were utilized to assess the relationships between baseline FI-CHG levels and cumulative FI-CHG exposure during follow-up with the incidence of CMM. Restricted cubic spline analyses were applied to investigate potential dose–response relationships, while subgroup analyses were implemented to examine possible effect modification.

**Result:**

During a maximum follow-up of 8.0 years, CMM occurred in 1,666 participants (19.5%). The risk of CMM increased progressively across higher quartiles of the FI-CHG index. In receiver operating characteristic analyses, the FI-CHG index demonstrated a slightly better predictive performance [area under the curve (AUC) = 0.692] compared with FI or CHG alone. Longitudinal analyses further showed that participants with persistently high FI-CHG levels and those in the top tertile of cumulative FI-CHG exposure exhibited the greatest risk of developing CMM.

**Conclusion:**

Our study demonstrated that higher baseline FI-CHG and cumulative FI-CHG exposure were significantly associated with increased risk of CMM. By integrating metabolic and functional indicators, the FI-CHG index may serve as a relatively robust metric with potential clinical prospects for risk stratification of chronic multimorbidity. It may help improve early risk identification and provide evidence for preventive strategies targeting cardiometabolic health.

## Graphical abstract

**Figure:**
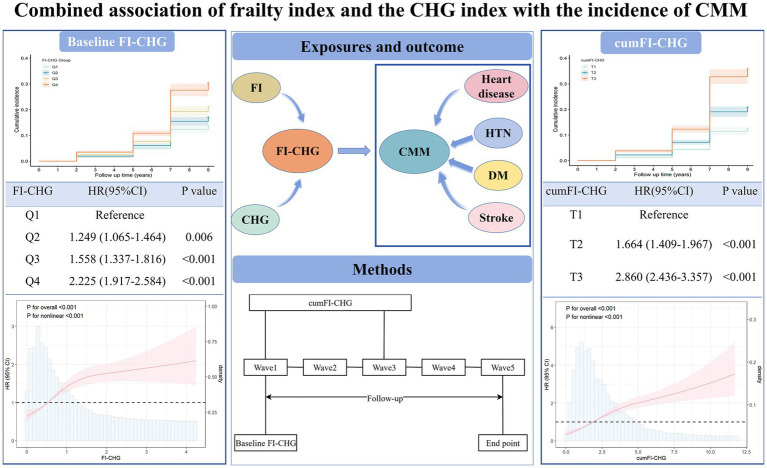

## 1 Introduction

As the global population ages rapidly, multimorbidity has become a significant public health challenge. Cardiometabolic multimorbidity (CMM), a highly prevalent and clinically significant subtype, is defined by coexisting cardiometabolic diseases (≥2 CMDs), including hypertension, type 2 diabetes mellitus (T2DM), stroke, and heart disease (1, 2). Data from a nationally representative Canadian cohort indicates that among individuals diagnosed with cardiovascular disease, diabetes mellitus, or stroke, the prevalence of at least one additional cardiometabolic condition was 32.2, 22.0, and 48.4%, respectively (3). Importantly, CMM is related to a substantially greater risk of all-cause mortality and a pronounced decline in life expectancy compared with having a single cardiometabolic disease (4). Furthermore, CMM has been consistently associated with functional disability, accelerated cognitive decline, incident dementia, and major depressive disorder (5–7). Thus, early identification of modifiable risk factors and the implementation of timely, evidence-based interventions are crucial to facilitate healthy aging and alleviate the growing burden on healthcare systems.

Insulin resistance (IR) represents a key pathophysiological mechanism underlying many cardiometabolic disorders. It contributes to the development of obesity, dyslipidemia, endothelial dysfunction, and chronic low-grade inflammation, processes that collectively promote atherosclerosis and subsequent cardiovascular disease (8). The triglyceride-glucose (TyG) index, a widely validated surrogate IR marker, has demonstrated strong predictive value for incident cardiovascular disease (CVD) and adverse cardiovascular outcomes. More recently, the cholesterol-high-density lipoprotein-glucose (CHG) index has been suggested as a potential composite biomarker reflecting systemic metabolic dysregulation. Compared with the TyG index, the CHG index shows higher diagnostic sensitivity and specificity for T2DM while maintaining comparable discriminatory capability for predicting CVD risk (9, 10). Against the backdrop of population aging and its associated health challenges, frailty has emerged as a key geriatric phenotype requiring systematic assessment. Frailty is a dynamic, multisystem clinical condition marked by progressive depletion of physiological reserves and elevated susceptibility to adverse health outcomes (11, 12). It reflects a complex state of increased vulnerability resulting from the accumulation of deficits across multiple domains, including physical, cognitive, and psychological functioning. The frailty index (FI), originally proposed by Rockwood et al., is a well-validated quantitative measure of biological aging and cumulative health deficit burden. It is computed as the ratio of existing health deficits (derived from a standardized set of age-related clinical, functional, cognitive, and psychological variables) to the total number of deficits measured for an individual (12). A growing body of evidence indicates that a greater FI is independently related to elevated risks of falls (13), cardiovascular events (14, 15), chronic liver disease (16), and all-cause mortality. Importantly, emerging research suggests that frailty may be partially reversible, with targeted interventions demonstrating potential to attenuate or even reverse frailty and thereby reduce related health risks (17–19).

Although physiological frailty and metabolic dysfunction frequently coexist in older adults, their combined and cumulative effects on the development of CMM remain insufficiently understood. To better capture the joint contribution of metabolic dysregulation and biological aging to CMM risk, we developed the FI-CHG index by integrating the FI with the CHG index. Using the China Health and Retirement Longitudinal Study (CHARLS), this research explored the associations of both baseline FI-CHG levels and long-term cumulative FI-CHG exposure with incident CMM. Restricted cubic spline (RCS) analyses, multivariable Cox proportional hazards models, and prespecified subgroup analyses were employed to comprehensively evaluate these relationships. By elucidating the combined influence of frailty and metabolic dysregulation on the development of CMM, our findings provide a basis for more comprehensive and clinically actionable strategies for risk stratification and prevention.

## 2 Methods

### 2.1 Data collection

**Figure 1:**
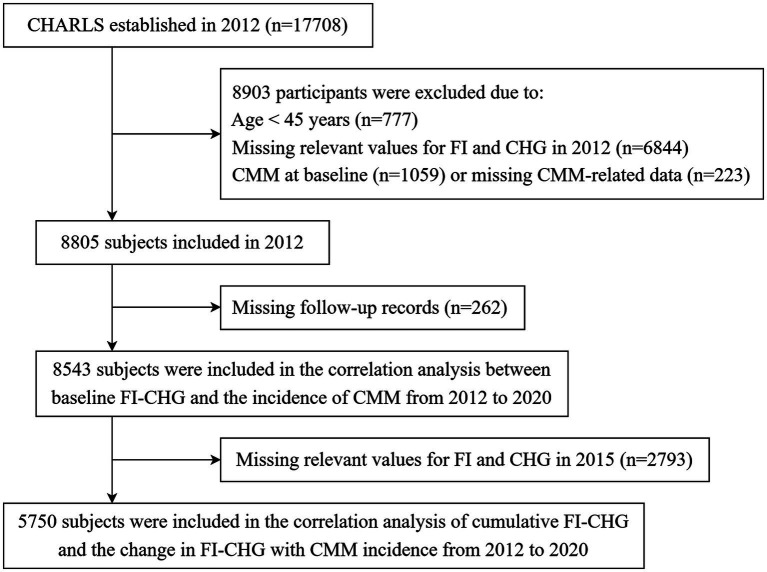
Flowchart of the study participants selection.

CHARLS, a nationally representative cohort established in 2012, was employed to investigate the health and aging of Chinese middle-aged and elderly individuals (≥45 years). Comprehensive data regarding the study design and sampling procedures has been described previously. Among the 17,708 participants recruited at baseline (2011-2012). 8,543 subjects were incorporated in the baseline FI-CHG and incident CMM analysis (2012–2020). Individuals aged <45 years, who had missing data on age, baseline CHG index, or FI, had pre-existing CMM or were lost to follow-up, were excluded (*n* = 9,165). For the cumulative FI-CHG analysis, 5,750 individuals with full FI and CHG measurements at waves 1 and 3 were incorporated. Figure 1 presents the detailed participant selection process.

### 2.2 Assessment of FI-CHG index

Frailty was assessed using a 28-item frailty index constructed from CHARLS data according to the deficit accumulation model. The index includes variables spanning comorbidities (excluding hypertension, diabetes, heart disease, and stroke), physical functioning, disability, depressive symptoms, and cognitive function. Each item was scored as 1 (deficit present) or 0 (deficit absent), except for the cognition item (item 28), which was scored on a continuous scale varying between 0 and 1. The FI score was computed as the sum of all item scores divided by 28, yielding a value between 0 and 1, with higher values indicating greater frailty. Missing data were handled according to established procedures. The FI was calculated only when no more than six of the 28 items (≤20%) were missing; otherwise, the score was considered missing. For eligible participants, missing items were imputed using the mean value of the participant’s non-missing items (20, 21).

The CHG index was calculated according to previously published methods as: CHG = Ln[TC_mg/dL_ × FBG_mg/dL_ / (2 × HDL_mg/dL_)] (9, 10). The combined FI-CHG metric was defined as the product of FI and CHG (FI × CHG), consistent with epidemiological approaches that use multiplicative terms to capture the joint effect of metabolic dysregulation and functional deficits (22, 23). To assess long-term exposure, the cumulative FI-CHG (cumFI-CHG) was computed as follows: cumFI-CHG = (FI-CHG _wave1_ + FI-CHG _wave3_) / 2 × time (2015–2012).

### 2.3 Ascertainment of CMM

The primary outcome was incident CMM, defined as the coexistence of ≥2 of the following conditions: hypertension, diabetes, CVD, or stroke. Hypertension was identified according to self-reported clinical diagnosis, antihypertensive drug use, diastolic blood pressure (DBP) ≥ 90 mmHg, or systolic blood pressure (SBP) ≥ 140 mmHg (24). Diabetes was defined by glycated hemoglobin (HbA1c) ≥ 6.5%, fasting blood glucose (FBG) ≥ 126 mg/dL, self-reported clinical diagnosis, or hypoglycemic medication use. Prediabetes was defined as 5.7–6.4% HbA1c or 100–125 mg/dL FBG, while participants without diabetes or prediabetes were classified as having normal glucose regulation (NGR) (24). Heart disease and stroke were identified in accordance with self-reported clinical diagnosis at baseline or during follow-up, or cardiovascular drug use (18, 25). During each survey round, participants were queried whether they had ever been diagnosed with severe cardiac diseases including myocardial infarction, angina pectoris, coronary heart disease, congestive heart failure and other clinically significant heart disorders, as well as stroke. For participants who developed CMM during follow-up, the event time was defined as the interval from baseline to the time at which a participant was diagnosed with a second distinct cardiometabolic disease. For those without incident CMM, follow-up time was computed from baseline to the date of the last completed survey.

### 2.4 Covariates

Baseline and follow-up data were collected through standardized interviewer-administered questionnaires, physical examinations, and fasting laboratory assessments. Demographic and lifestyle variables included age, sex, educational attainment, place of residence, alcohol intake and smoking behavior. Self-reported medical history and medication use were recorded. Fasting venous blood specimens were used to assess FBG, total cholesterol (TC), triglycerides (TG), low-density lipoprotein cholesterol (LDL-C), high-density lipoprotein cholesterol (HDL-C), serum creatinine, C-reactive protein (CRP), and uric acid (UA). Dyslipidemia was defined as a self-reported clinical diagnosis, lipid-lowering medication use, or the presence of any of the following lipid abnormalities: LDL-C ≥ 160 mg/dL, TG ≥ 150 mg/dL, or TC ≥ 240 mg/dL (26).

### 2.5 Statistical analysis

Continuous variables with normal distribution are expressed as mean±standard deviation (SD) and were compared using one-way ANOVA. Those with non-normal distribution are reported as median with interquartile range (IQR) and were compared through Kruskal-Wallis test. Categorical variables are presented as frequencies (percentages) and compared through Fisher’s exact test or χ^2^ test, as appropriate. Missing data were handled with multiple imputation for variables with minimal missingness, whereas individuals with extensive missing data were excluded.

All subjects were categorized into four groups according to baseline FI-CHG quartiles (Q1-Q4) and into four FI × CHG classes. Quartiles (Q1-Q4) represented increasing levels of baseline FI-CHG. The FI × CHG classes were defined using median splits of FI and CHG: Class 1 (low FI/low CHG), Class 2 (low FI/high CHG), Class 3 (high FI/low CHG), and Class 4 (high FI/high CHG).

To characterize longitudinal patterns of FI-CHG, K-means clustering was utilized to identify trajectories from Wave 1 to Wave 3. Utilizing the elbow approach on the within-cluster sum of squares (WSS), *K* = 3 was identified as the optimal number of clusters. All FI-CHG values were standardized prior to clustering, as illustrated in Supplementary Figure S1. Participants were subsequently assigned to three trajectory clusters: Cluster 1 (low-stable: 0.36 → 0.45), Cluster 2 (moderate-increasing: 0.94 → 1.20), and Cluster 3 (high-increasing: 1.96 → 2.21), where values represent the mean FI-CHG scores in 2012 and 2015.

Associations between FI-CHG and incident CMM risk were examined using multivariable Cox regression models. Three models were developed: a crude model without covariate adjustment; Model 1 adjusted for age, gender, residence, education level, marital status, alcohol intake and smoking behavior; and Model 2 further adjusted for dyslipidemia, CRP, UA, and HbA1c. RCS models analyzed linear and nonlinear dose–response relationships between FI-CHG and CMM risk. Four knots at the 5th, 35th, 65th and 95th percentiles were set via the rms package following standard spline specifications. Cumulative incidence of CMM across FI-CHG groups was estimated using Kaplan–Meier curves and compared with the log-rank test. Predictive performance was evaluated using receiver operating characteristic (ROC) analyses, as well as integrated discrimination improvement (IDI) and net reclassification improvement (NRI).

Stratified analyses were conducted according to age, gender, marital status, alcohol intake, smoking behavior, body mass index (BMI), estimated glomerular filtration rate (eGFR), and history of chronic disease to assess potential effect modification in the associations of baseline FI-CHG and cumulative FI-CHG with CMM risk. Effect modification was assessed using exposure-by-subgroup interaction terms. Interaction *p*-values were derived from Wald tests for binary variables and likelihood ratio tests for multicategory variables. Sensitivity analyses were carried out to evaluate the reliability of the findings, including: (1) a complete-case analysis without multiple imputation; (2) exclusion of individuals with baseline chronic diseases; (3) exclusion of individuals receiving lipid-lowering or glucose-lowering medications; (4) exclusion of cases developing CMM within the first 2 years of follow-up; (5) E-value assessment of unmeasured confounding; and (6) two-cluster grouping of FI-CHG trajectories via K-means clustering determined by the elbow method. All statistical analyses were implemented using Stata v17 and R v4.2. *p* < 0.05 was considered statistically significant.

## 3 Results

### 3.1 Baseline features of study subjects

Overall, 8,543 subjects (median age 57.0 [51.0, 65.0] years; 52.8% female) were incorporated. During a maximum follow-up of 96 months, CMM occurred in 1,666 participants (19.5%). Baseline characteristics according to CMM status are presented in Supplementary Table S1. Participants who developed CMM showed significantly different baseline FI distributions and had higher CHG index and FI-CHG scores than those who remained free of CMM (all *p* < 0.001).

Participants were further stratified into quartiles according to baseline FI-CHG levels (Q1-Q4), and their baseline characteristics are presented in Table 1. Most characteristics differed significantly across FI-CHG quartiles. Participants in higher FI-CHG quartiles were generally older and more likely to be female. Levels of FBG, HbA1c, CRP, TG, TC, FI, and FI-CHG tended to be higher across quartiles. The prevalence of hypertension, diabetes, heart disease, stroke, dyslipidemia, and CMM also increased across FI-CHG quartiles (all *p* < 0.05).

**Table 1:** Baseline characteristics of participants stratified by FI-CHG quartiles.

Characteristics	Overall (8,543)	Q1 (2,136)	Q2 (2,136)	Q3 (2,136)	Q4 (2,135)	*P*
Age	57.0 (51.0, 65.0)	55.0 (49.0, 61.0)	56.0 (50.0, 63.0)	57.0 (51.0, 64.0)	61.0 (55.0, 69.0)	<0.001
Gender						<0.001
Female	4,507 (52.8)	989 (46.3)	1,007 (47.1)	1,189 (55.7)	1,322 (61.9)	
Male	4,036 (47.2)	1,147 (53.7)	1,129 (52.9)	947 (44.3)	813 (38.1)	
Education level						<0.001
Illiterate	927 (10.9)	322 (15.1)	293 (13.7)	214 (10.0)	98 (4.6)	
Sishu/homeschool/elementary school	2,483 (29.1)	480 (22.5)	489 (22.9)	613 (28.7)	901 (42.2)	
Middle school	1,730 (20.3)	540 (25.3)	525 (24.6)	409 (19.1)	256 (12.0)	
High school and above	3,403 (39.8)	794 (37.2)	829 (38.8)	900 (42.1)	880 (41.2)	
Marital status						<0.001
Non-married	7,566 (88.6)	1,983 (92.8)	1,944 (91.0)	1,875 (87.8)	1,764 (82.6)	
Married	977 (11.4)	153 (7.2)	192 (9.0)	261 (12.2)	371 (17.4)	
Location						<0.001
Village	7,053 (82.6)	1,685 (78.9)	1,691 (79.2)	1,781 (83.4)	1,896 (88.8)	
City/town	1,490 (17.4)	451 (21.1)	445 (20.8)	355 (16.6)	239 (11.2)	
Smoking status						<0.001
No	5,178 (60.6)	1,261 (59.0)	1,216 (56.9)	1,319 (61.8)	1,382 (64.7)	
Yes	3,365 (39.4)	875 (41.0)	920 (43.1)	817 (38.2)	753 (35.3)	
Drinking status						0.036
No	5,149 (60.3)	1,310 (61.3)	1,235 (57.8)	1,284 (60.1)	1,320 (61.8)	
Yes	3,394 (39.7)	826 (38.7)	901 (42.2)	852 (39.9)	815 (38.2)	
SBP (mmHg)	126.0 (113.7, 141.3)	126.3 (114.3, 141.4)	125.7 (113.7, 140.4)	126.0 (113.3, 141.7)	126.7 (113.3, 141.8)	0.713
DBP (mmHg)	74.3 (66.7, 83.3)	75.7 (67.3, 84.7)	74.7 (66.3, 84.0)	74.3 (67.0, 82.7)	73.7 (65.7, 82.0)	<0.001
BMI (Kg/m2)	23.1 (20.8, 25.7)	23.2 (21.0, 25.5)	23.0 (20.7, 25.6)	23.3 (20.9, 25.9)	22.8 (20.5, 25.7)	0.031
FBG(mg/dl)	101.7 (94.1, 111.2)	101.5 (94.0, 110.7)	101.2 (94.0, 110.0)	101.7 (94.0, 111.2)	102.4 (94.7, 113.0)	0.004
HbA1c (%)	5.1 (4.9, 5.4)	5.1 (4.9, 5.4)	5.1 (4.9, 5.4)	5.1 (4.9, 5.4)	5.2 (4.9, 5.5)	<0.001
UA (mg/dl)	4.3 (3.6, 5.1)	4.4 (3.6, 5.2)	4.3 (3.6, 5.2)	4.3 (3.6, 5.1)	4.2 (3.5, 5.0)	<0.001
CRP (mg/dl)	1.0 (0.5, 2.1)	0.9 (0.5, 1.9)	0.9 (0.5, 1.9)	1.0 (0.5, 2.2)	1.1 (0.6, 2.4)	<0.001
eGFR(mL/min/1.73 m2)	95.0 (84.6, 102.5)	96.6 (86.8, 103.8)	95.7 (84.8, 102.9)	95.3 (84.9, 102.7)	92.7 (82.3, 100.5)	<0.001
TG (mg/dl)	102.7 (73.5, 148.7)	97.3 (69.9, 148.7)	100.9 (73.5, 146.0)	103.5 (73.5, 146.9)	107.1 (77.9, 154.0)	<0.001
TC (mg/dl)	191.0 (167.4, 215.7)	188.7 (166.2, 213.4)	190.6 (166.6, 215.3)	191.4 (168.9, 216.1)	192.5 (168.2, 219.2)	0.008
LDL (mg/dl)	115.2 (94.3, 137.8)	114.4 (94.7, 135.7)	115.2 (95.1, 137.6)	115.6 (93.9, 138.4)	115.2 (93.6, 139.2)	0.689
HDL (mg/dl)	49.9 (41.0, 60.3)	50.3 (40.2, 60.7)	49.9 (41.0, 60.3)	49.9 (41.4, 60.7)	49.5 (40.6, 60.1)	0.765
FI	0.1 (0.1, 0.2)	0.0 (0.0, 0.0)	0.1 (0.1, 0.1)	0.1 (0.1, 0.2)	0.3 (0.2, 0.3)	<0.001
CHG	5.3 (5.0, 5.5)	5.2 (5.0, 5.5)	5.3 (5.0, 5.5)	5.3 (5.0, 5.5)	5.3 (5.0, 5.6)	0.003
FI-CHG	0.5 (0.3, 1.0)	0.1 (0.1, 0.2)	0.4 (0.3, 0.5)	0.7 (0.6, 0.8)	1.4 (1.1, 1.8)	<0.001
Hypertension						<0.001
No	6,859 (80.3)	1,811 (84.8)	1,757 (82.3)	1,676 (78.5)	1,615 (75.6)	
Yes	1,684 (19.7)	325 (15.2)	379 (17.7)	460 (21.5)	520 (24.4)	
Diabetes						<0.001
No	8,099 (94.8)	2,040 (95.5)	2,043 (95.6)	2029 (95.0)	1987 (93.1)	
Yes	444 (5.2)	96 (4.5)	93 (4.4)	107 (5.0)	148 (6.9)	
Heart disease						<0.001
No	8,031 (94.0)	2067 (96.8)	2042 (95.6)	2020 (94.6)	1902 (89.1)	
Yes	512 (6.0)	69 (3.2)	94 (4.4)	116 (5.4)	233 (10.9)	
Stroke						<0.001
No	8,477 (99.2)	2,130 (99.7)	2,126 (99.5)	2,119 (99.2)	2,102 (98.5)	
Yes	66 (0.8)	6 (0.3)	10 (0.5)	17 (0.8)	33 (1.5)	
Dyslipidemia						<0.001
No	7,862 (92.0)	2,025 (94.8)	1,971 (92.3)	1,954 (91.5)	1,912 (89.6)	
Yes	681 (8.0)	111 (5.2)	165 (7.7)	182 (8.5)	223 (10.4)	
Antihypertensive drugs						<0.001
No	7,327 (85.8)	1,920 (89.9)	1,874 (87.7)	1,796 (84.1)	1,737 (81.4)	
Yes	1,216 (14.2)	216 (10.1)	262 (12.3)	340 (15.9)	398 (18.6)	
Hypoglycemic drugs						<0.001
No	8,392 (98.2)	2,116 (99.1)	2,107 (98.6)	2,090 (97.8)	2,079 (97.4)	
Yes	151 (1.8)	20 (0.9)	29 (1.4)	46 (2.2)	56 (2.6)	
Lipid-lowering drugs						<0.001
No	8,232 (96.4)	2,090 (97.8)	2,072 (97.0)	2,048 (95.9)	2,022 (94.7)	
Yes	311 (3.6)	46 (2.2)	64 (3.0)	88 (4.1)	113 (5.3)	
CMM outcome						<0.001
No	6,877 (80.5)	1,864 (87.3)	1,790 (83.8)	1,710 (80.1)	1,513 (70.9)	
Yes	1,666 (19.5)	272 (12.7)	346 (16.2)	426 (19.9)	622 (29.1)	

FI, frailty index; CHG, Cholesterol, High-Density Lipoprotein, Glucose index; SBP, systolic blood pressure; DBP, diastolic blood pressure; BMI, body mass index; FBG, fasting blood glucose; HbA1c, hemoglobin A1c; UA, uric acid; CRP, C-reactive protein; TG, triglycerides; TC, total cholesterol; LDL, low-density Lipoprotein cholesterol; HDL, high-density lipoprotein cholesterol; CMM, cardiometabolic multimorbidity.

### 3.2 Association between baseline FI, CHG, FI&CHG, FI-CHG with CMM risk

**Figure 2:**
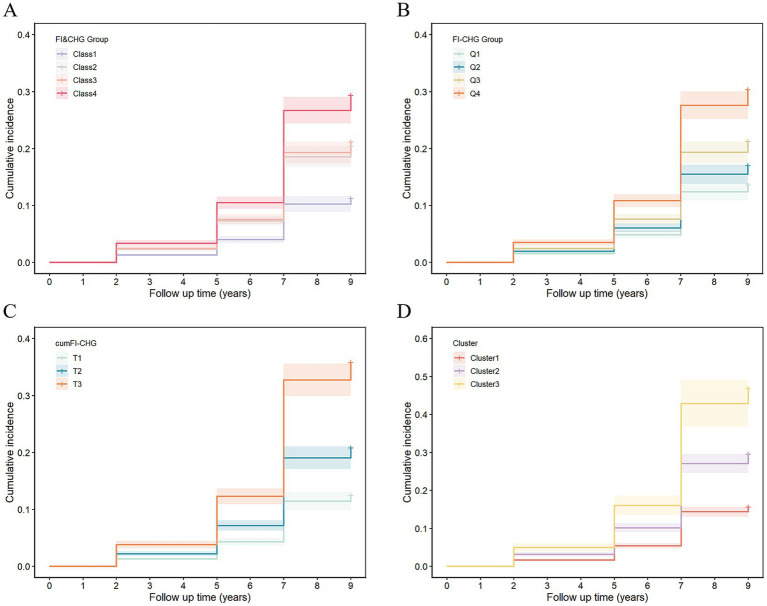
Kaplan–Meier curves for cumulative incidence of CMM outcomes across baseline FI&CHG, FI-CHG, cumFI-CHG based groups and longitudinal clusters. **(A)** Median stratification for FI and CHG; **(B)** FI-CHG quartile grouping; **(C)** Cumulative FI-CHG tertile grouping; **(D)** FI-CHG trajectory clusters. Model 2: Age, gender, location, education, marital status, smoking, drinking, dyslipidemia, CRP, UA, and HbA1c were adjusted. CMM, cardiometabolic multimorbidity; FI, frailty index; CHG, Cholesterol, High-Density Lipoprotein, Glucose index; cumFI-CHG, cumulative FI-CHG.

Kaplan–Meier survival curves estimating the cumulative incidence of CMM according to joint FI and CHG status and FI-CHG quartiles are shown in Figure 2. Participants with both high FI and high CHG, as well as those in the top FI-CHG quartile, showed the highest cumulative incidence of CMM compared with their respective reference groups (log-rank *p* < 0.001). The cumulative incidence of CMM increased progressively across FI-CHG quartiles: 12.7% in Q1, 16.2% in Q2, 19.9% in Q3, and 29.1% in Q4, indicating a graded association between higher FI-CHG levels and greater CMM risk.

Multivariable Cox regression models were utilized to examine the relationships among FI, CHG, and CMM. In the crude model, both high FI (HR = 1.596, 95%CI:1.442–1.767) and high CHG (HR = 1.790, 95%CI:1.621–1.978) were related to a greater risk of CMM. These associations remained significant after multivariable adjustment. Utilizing the low FI/low CHG group (Class 1) as the reference, the greatest risk was noted among participants with both high FI and high CHG (HR = 2.602, 95%CI:2.212–3.061). Higher risks were also observed in the high FI/low CHG group (HR = 1.879, 95%CI:1.587–2.224) and the low FI/high CHG group (HR = 1.808, 95%CI:1.528–2.140) in the fully adjusted model (Table 2).

**Table 2:** Association between baseline FI, CHG, FI&CHG, FI-CHG, and CMM risk.

Variables	Crude model		Model 1		Model 2	
	HR (95%CI)	*P*	HR (95%CI)	*P*	HR (95%CI)	*P*
FI						
Low	Ref		Ref		Ref	
High	1.596 (1.442–1.767)	<0.001	1.614 (1.457–1.789)	<0.001	1.588 (1.433–1.761)	<0.001
CHG						
Low	Ref		Ref		Ref	
High	1.790 (1.621–1.978)	<0.001	1.795 (1.624–1.983)	<0.001	1.526 (1.375–1.694)	<0.001
FI&CHG						
Class 1	Ref		Ref		Ref	
Class 2	2.148 (1.822–2.533)	<0.001	2.138 (1.812–2.522)	<0.001	1.808 (1.528–2.140)	<0.001
Class 3	2.063 (1.746–2.437)	<0.001	1.928 (1.629–2.282)	<0.001	1.879 (1.587–2.224)	<0.001
Class 4	3.371 (2.879–3.946)	<0.001	3.136 (2.673–3.678)	<0.001	2.602 (2.212–3.061)	<0.001
FI-CHG						
Per 1 unit	1.580 (1.488–1.678)	<0.001	1.515 (1.419–1.618)	<0.001	1.452 (1.360–1.551)	<0.001
Quartile						
Q1	Ref		Ref		Ref	
Q2	1.290 (1.101–1.512)	0.002	1.275 (1.088–1.495)	0.003	1.249 (1.065–1.464)	0.006
Q3	1.638 (1.407–1.907)	<0.001	1.598 (1.371–1.863)	<0.001	1.558 (1.337–1.816)	<0.001
Q4	2.540 (2.203–2.930)	<0.001	2.382 (2.053–2.765)	<0.001	2.225 (1.917–2.584)	<0.001
P for trend		<0.001		<0.001		<0.001

Crude model: No covariates were adjusted.

Model 1: Age, gender, location, education, marital, smoking, and drinking were adjusted.

Model 2: dyslipidemia, CRP, UA, and HbA1c were further adjusted.

Class 1: FI ≤ median and CHG ≤ median; Class 2: FI ≤ median and CHG > median; Class 3: FI > median and CHG ≤ median; Class 4: FI > median and CHG > median.

A strong graded association was also found between the FI-CHG index and CMM risk. After full adjustment, each 1-unit rise in FI-CHG was related to a 45.2% greater risk of developing CMM (HR = 1.452; 95%CI:1.360–1.551). Compared to subjects in the lowest quartile (Q1), those in Q2, Q3, and Q4 exerted significantly greater risks of CMM in the fully adjusted model, with HRs of 1.249 (95%CI:1.065–1.464), 1.558 (95%CI:1.337–1.816), and 2.225 (95%CI:1.917–2.584), respectively (*P*_trend_ < 0.001). This dose–response association was consistent across all models.

**Figure 3:**
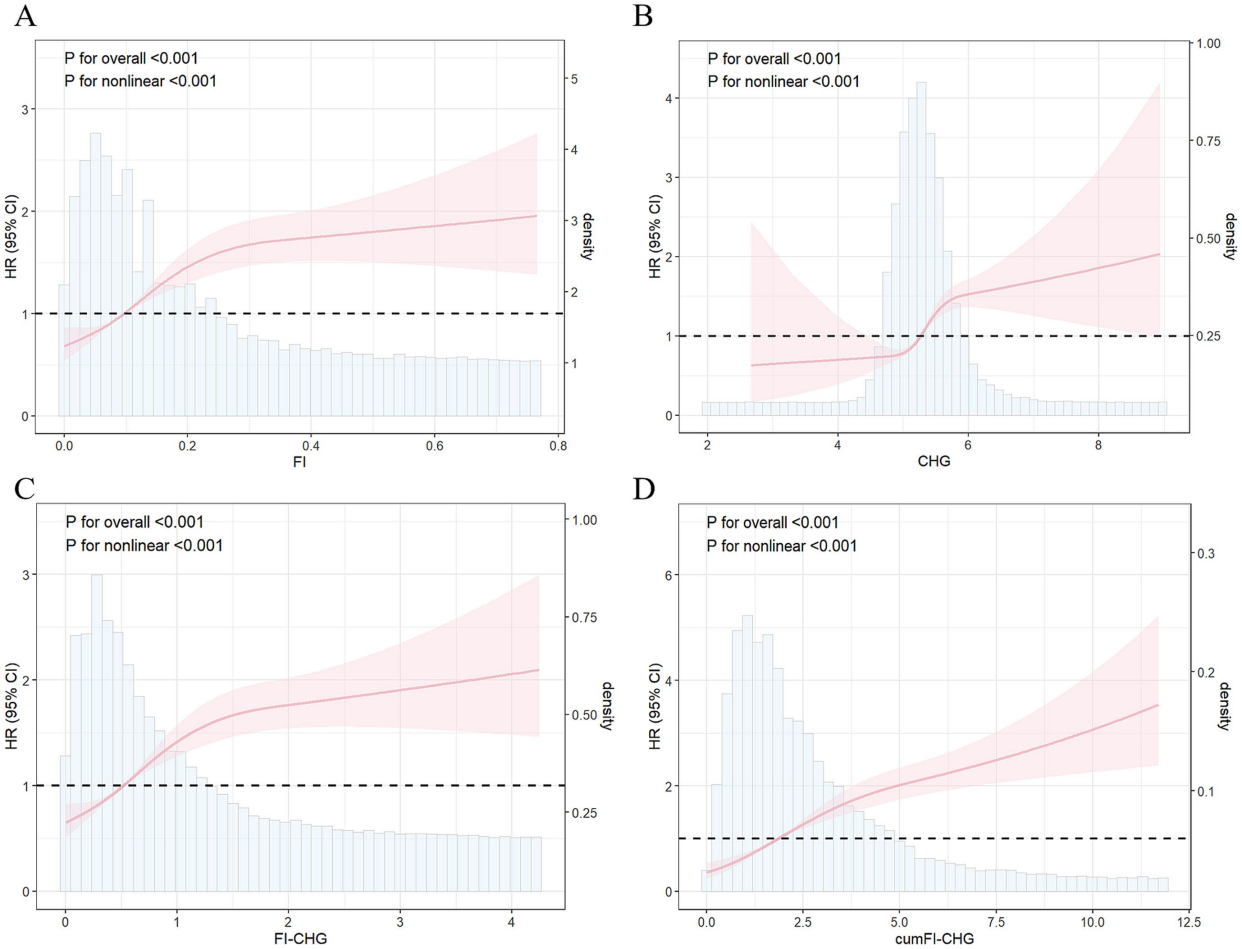
Restricted cubic spline analysis of the relationships among baseline FI, CHG, FI-CHG, cumFI-CHG, and the CMM risk. **(A)** FI; **(B)** CHG; **(C)** FI-CHG; **(D)** cumulative FI-CHG. Model 2: Age, gender, location, education, marital status, smoking, drinking, dyslipidemia, CRP, UA, and HbA1c were adjusted. CMM, cardiometabolic multimorbidity; FI, frailty index; CHG, Cholesterol, High-Density Lipoprotein, Glucose index; cumFI-CHG, cumulative FI-CHG.

RCS analyses adjusted for all covariates demonstrated significant nonlinear associations between FI, CHG, and FI-CHG with CMM risk (all *p* < 0.01 for overall association and nonlinearity; Figure 3). The risk of CMM increased progressively across the full range of each exposure.

### 3.3 Association between FI-CHG variability, cumulative FI-CHG, and the risk of CMM

K-means clustering was applied to classify longitudinal FI-CHG trajectories between 2012 and 2015 into three distinct patterns. Among the 5,750 participants with complete FI-CHG measurements at both time points, three clusters were identified: Cluster 3 (high-increasing), Cluster 2 (moderate-increasing), and Cluster 1 (low-stable). Baseline characteristics across trajectory clusters are summarized in Supplementary Tables S2, S3.

In the crude model, participants in Cluster 3 exerted a substantially greater risk of developing CMM compared to those in Cluster 1 (HR = 3.393; 95%CI:2.894–3.979). After full multivariable adjustment, both Cluster 2 and Cluster 3 remained significantly related to CMM risk, with HRs of 1.886 (95%CI:1.661–2.143) and 2.985 (95%CI:2.521–3.534), respectively.

Tertile-based analyses further demonstrated a remarkable dose–response association between cumulative FI-CHG (cumFI-CHG) and CMM risk. After full adjustment, each 1-unit rise in cumFI-CHG was related to a 22.8% greater risk of CMM (HR = 1.228; 95%CI:1.195–1.261). Compared to the lowest tertile (T1), subjects in T2 and T3 had 1.66-fold (95%CI:1.409–1.967) and 2.86-fold (95%CI:2.436–3.357) greater risks of CMM, respectively (Table 3). RCS analyses further supported a nonlinear but monotonic increase in CMM risk across the distribution of cumFI-CHG (Figure 3). Overall, the highest CMM risk was consistently observed among participants with the greatest FI-CHG exposure, whether defined by longitudinal trajectories, cumulative tertiles, or baseline quartiles, after adjustment for potential confounders.

**Table 3:** Association between the variation of FI-CHG and cumulative FI-CHG and CMM risk.

Variables	Crude model		Model 1		Model 2	
	HR (95%CI)	*P*	HR (95%CI)	*P*	HR (95%CI)	*P*
K-means Group						
Cluster1	Ref		Ref		Ref	
Cluster2	2.022 (1.787–2.288)	<0.001	1.961 (1.727–2.228)	<0.001	1.886 (1.661–2.143)	<0.001
Cluster3	3.393 (2.894–3.979)	<0.001	3.151 (2.662–3.731)	<0.001	2.985 (2.521–3.534)	<0.001
CumFI-CHG						
Per 1 unit	1.255 (1.225–1.287)	<0.001	1.241 (1.208–1.275)	<0.001	1.228 (1.195–1.261)	<0.001
Tertile						
T1	Ref		Ref		Ref	
T2	1.758 (1.490–2.074)	<0.001	1.732 (1.466–2.046)	<0.001	1.664 (1.409–1.967)	<0.001
T3	3.185 (2.735–3.710)	<0.001	3.041 (2.592–3.568)	<0.001	2.860 (2.436–3.357)	<0.001
P for trend		<0.001		<0.001		<0.001

Crude model: No covariates were adjusted.

Model 1: Age, gender, location, education, marital, smoking, and drinking were adjusted.

Model 2: dyslipidemia, CRP, UA, and HbA1c were further adjusted.

### 3.4 Predictive performance of the FI-CHG index for CMM risk

ROC analysis showed that FI-CHG had an AUC of 0.692 (95% CI: 0.655–0.729) for predicting CMM, compared with 0.690 (95% CI: 0.653–0.727) for FI and 0.670 (95% CI: 0.632–0.708) for CHG (Supplementary Figure S2). When added to the baseline model, FI-CHG yielded a cNRI of 0.148 (95% CI: 0.083–0.223; *p* < 0.001) and an IDI of 0.006 (95% CI: 0.001–0.011; *p* = 0.004), while the corresponding values for FI were 0.152 (95% CI: 0.072–0.214; *p* < 0.001) and 0.005 (95% CI: 0.001–0.009; *p* = 0.004), respectively. CHG alone showed weaker reclassification performance and a non-significant IDI (Supplementary Table S4).

### 3.5 Subgroup analyses

**Figure 4:**
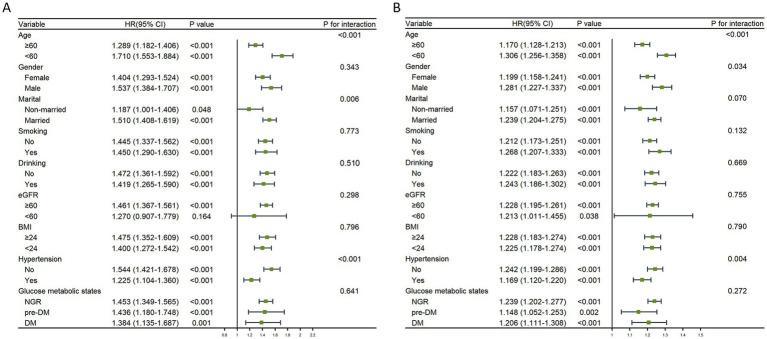
Subgroup analysis of the relationship between FI-CHG and the CMM risk. **(A)** Subgroup analysis for baseline FI-CHG; **(B)** Subgroup analysis for cumFI-CHG. Model 2: Age, gender, location, education, marital status, smoking, drinking, dyslipidemia, CRP, UA, and HbA1c were adjusted. FI, frailty index; CHG, Cholesterol, High-Density Lipoprotein, Glucose index; cumFI-CHG, cumulative FI-CHG; CMM, cardiometabolic multimorbidity; eGFR, estimated glomerular filtration rate; BMI, body mass index; NGR, normal glucose regulation; pre-DM, prediabetes; DM, diabetes mellitus; HR, hazard ratio; CI, confidence interval.

Subgroup analyses evaluating the relationship between FI-CHG and CMM risk across categories of age, gender, marital status, alcohol intake, smoking behavior, eGFR, BMI, and history of chronic disorder are presented in Figure 4. Significant effect modification was noted for age, marital status, and hypertension history in the analyses of baseline FI-CHG, whereas no significant interactions were detected for the remaining subgroups. The association between FI-CHG and CMM risk was strongest among participants aged ≤75 years (Supplementary Figure S3). For cumulative FI-CHG, significant interactions were observed for age, gender, and hypertension history, with a similarly stronger association among individuals aged ≤75 years (Supplementary Figure S4).

### 3.6 Sensitivity analyses

The results of sensitivity analyses remained consistent in the complete-case cohort without multiple imputation (Supplementary Table S5), among participants without baseline chronic disorders (diabetes, hypertension, heart disease, or stroke; Supplementary Table S6), among those not receiving lipid-lowering or glucose-lowering medications at baseline (Supplementary Table S7), and among participants excluding those with incident CMM within the first 2 years of follow-up (Supplementary Table S8). E-values calculated from all models ranged from 1.680 to 6.243, suggesting that only a strong unmeasured confounder could fully explain the observed associations (Supplementary Table S9). Ultimately, applying the double-cluster FI-CHG trajectory grouping method—determined via the elbow method—yielded consistent results, reinforcing the robustness of the conclusion (Supplementary Table S10). Collectively, these analyses confirmed the reliability of the observed relationship between elevated FI-CHG and increased CMM risk.

## 4 Discussion

This study provides novel, large-scale evidence linking the FI-CHG index to CMM risk among middle-aged and elderly Chinese individuals. Both baseline and cumulative FI-CHG exposure were independently related to a greater risk of CMM. These associations remained robust following comprehensive adjustment for demographic, behavioral, clinical, and socioeconomic confounders. RCS analyses further indicated a significant positive nonlinear relationship between FI-CHG and CMM risk across its full distribution. Subgroup analyses indicated that the relationship was largely consistent across strata defined by age, sex, marital status, renal function, BMI, and chronic disease history, although significant effect modification was observed for age, marital status, and hypertension. Moreover, three sensitivity analyses produced results consistent with the primary findings. Collectively, these results support the FI-CHG index as a robust and integrative biomarker for CMM risk stratification in this population.

The CHG index is a recently proposed biomarker derived from TC, HDL-C, and FBG. It was first introduced by Mansoori et al., who reported that CHG outperformed the established TyG index in discriminating T2DM individuals in comparative diagnostic accuracy analyses. Subsequent studies have further demonstrated positive associations between CHG levels and CVD risk (27, 28). For example, a cohort analysis of 6,249 participants from the CHARLS study showed that individuals in the highest CHG category had a significantly greater risk of incident cardiovascular events. In addition, CHG exhibited predictive performance for CVD risk comparable to that of the TyG index. Evidence from the Kailuan cohort further indicates that, compared to other IR markers [e.g., the atherogenic index of plasma (AIP) and TyG], the CHG index has better predictive value for both incident CVD and all-cause mortality (29).

Consistent with this growing body of evidence, our findings demonstrate a robust dose–response association between CHG levels and CMM risk. One important advantage of the CHG index lies in its integration of three key metabolic parameters, allowing it to capture interconnected disturbances in lipid metabolism, glycemic regulation, and cardiovascular health. High LDL-C and TC levels are known risk factors for increased cardiovascular mortality, whereas higher HDL-C concentrations exert protective effects against CVD (28, 30). By incorporating both atherogenic and anti-atherogenic lipid components alongside glycemic status, the CHG index reflects the balance of metabolic factors that contribute to cardiovascular pathophysiology. Furthermore, CHG represents a simple and cost-effective metric that can be readily calculated using routinely available fasting blood tests. Unlike other indices of insulin resistance, it does not require measurements of insulin or HOMA-IR. This practicality enhances its applicability in both epidemiological and clinical settings for the integrated assessment of insulin resistance and dyslipidemia.

The frailty index represents another important determinant of cardiovascular outcomes (31, 32). Previous reports have suggested that worsening physical frailty is related to a greater risk of CVD, whereas improvements in physical function are linked to reduced CVD risk. For instance, He and co-workers demonstrated that both frailty and pre-frailty are consistently related to higher risks of all-cause and CVD-specific mortality among T2DM individuals (18). Accumulating evidence further suggests that frailty contributes to a greater incidence of cardiometabolic disorders and comorbid conditions and acts as a promising predictor of CMM progression (33). After independently evaluating CHG and FI levels, our combined subgroup analysis demonstrated a markedly higher risk of cardiometabolic multimorbidity among individuals with concurrently elevated levels of both markers, suggesting a potential synergistic interaction (15, 34). To better capture the joint influence of metabolic dysfunction and physiological decline, we integrated the CHG index with the frailty index to develop FI-CHG as a combined risk marker, aiming to facilitate simplified risk stratification and early identification of cardiometabolic multimorbidity. In our analysis, higher baseline FI-CHG levels, greater cumulative FI-CHG exposure, and persistent high-stable FI-CHG trajectories were each independently related to a greater risk of CMM. These associations remained robust across multiple sensitivity analyses and across diverse population subgroups, including older adults, individuals with diabetes, and those with pre-existing cardiovascular disease. Together, these findings support the FI-CHG index as a comprehensive risk marker capturing both cardiometabolic and aging-related vulnerability.

Recent studies have demonstrated that composite indices integrating frailty status with metabolic parameters can effectively improve disease risk stratification. For example, eGDR-FI, which combines the estimated glucose disposal rate (eGDR) with the FI, was shown in the CHARLS cohort to predict cardiovascular events and all-cause mortality better than either indicator alone (35). Similarly, TyG-FI, integrating the TyG index with the FI, was closely associated with incident CMM (36). In addition, AIP-FI, derived from the combination of the AIP and FI, was significantly associated with increased risks of cardiovascular disease, stroke, and heart disease (23). These findings suggest that, compared with single-dimensional indicators, integrating metabolic abnormalities with frailty status may provide more comprehensive prognostic information. Compared with TyG-FI and AIP-FI, the FI-CHG constructed in the present study combines frailty phenotype with a composite metabolic parameter derived from TC, FBG, and HDL-C, thereby capturing multiple metabolic abnormalities, including dysglycemia, lipid disorders, and decline in physiological reserve. Moreover, FI-CHG may partially reflect the balance between atherogenic lipid burden and protective lipid components. This multidimensional integrative characteristic may therefore better characterize the metabolic vulnerability associated with frailty.

Our results highlight the pathophysiological synergy between metabolic dysfunction and multisystem physiological decline in driving cardiovascular risk. Moreover, the integrative nature of the FI-CHG index may facilitate more refined risk stratification and enable earlier identification of individuals at elevated risk for cardiometabolic comorbidities. The multidimensional structure and practical applicability of FI-CHG underscore its potential value in informing precision prevention strategies for cardiovascular disease. Dysregulation of glucose and lipid metabolism can promote systemic inflammation, marked by higher pro-inflammatory cytokine levels, which in turn contribute to endothelial dysfunction and accelerate atherosclerotic progression (37). In addition, hypercholesterolemia and hyperglycemia are potent drivers of oxidative stress. Insulin resistance further amplifies this oxidative burden by impairing antioxidant defense systems, reducing nitric oxide bioavailability, promoting endothelial dysfunction, and upregulating inflammatory mediators, thereby exacerbating vascular injury and atherosclerosis (8, 38). Frailty itself is also characterized by chronic low-grade inflammation, immunosenescence, and mitochondrial dysfunction. These processes can worsen endothelial injury, accelerate vascular aging, and further promote atherosclerotic development, ultimately increasing cardiovascular susceptibility (39). The convergence of these mechanisms offers a strong biological basis for the synergistic interaction between frailty and cardiometabolic dysregulation: insulin resistance amplifies the vascular and inflammatory vulnerabilities associated with frailty, while frailty intensifies the systemic consequences of metabolic stress.

This research has some notable methodological and conceptual strengths. To our knowledge, it is the first nationwide study to propose the FI-CHG index, a composite risk indicator that integrates metabolic dysregulation (CHG) and physiological decline (FI) to evaluate the risk of CMM. By simultaneously capturing metabolic abnormalities and functional deterioration, this integrated approach enables a more comprehensive assessment of susceptibility to cardiometabolic comorbidities. Second, rather than relying on a single measurement, we evaluated the longitudinal trajectory of FI-CHG to better characterize its temporal dynamics within the population. This approach provides a deeper understanding of long-term fluctuation patterns and strengthens the robustness of longitudinal analyses by capturing dynamic changes over time. Third, this research employed datasets from a large, nationally representative longitudinal cohort, thereby enhancing the reliability and generalizability of the findings. In addition, subgroup and interaction analyses not only identified population-specific associations but also highlighted the potential value of FI-CHG for more refined risk stratification. Collectively, these strengths underscore the novelty and potential translational relevance of FI-CHG as a biomarker for predicting CMM risk.

Nevertheless, a few limitations warrant acknowledgment. First, trajectory modeling was based on FI-CHG assessments from the first and third survey waves; inclusion of additional intermediate time points would improve temporal resolution and allow more precise characterization of longitudinal patterns. Second, incident heart disease and stroke were partially identified via self-reported diagnoses, which may introduce misclassification bias. Age-related cognitive decline among middle-aged and older adults may further cause recall bias and reduce data accuracy. However, this method has been widely adopted in large population-based cohort studies and validated for reliable identification of major cardiovascular events (18, 25). The English Longitudinal Study of Ageing (ELSA), with access to medical records for validation, documented a 77.5% concordance between self-reported CVD cases and verified medical records (40). Third, due to the observational nature of our study, causal inference cannot be established and reverse causation may exist. This concern is particularly relevant among older adults, in whom multimorbidity is highly prevalent and chronic conditions tend to mutually exacerbate frailty. However, our findings remained stable after excluding participants who developed endpoint events within the first 2 years of follow-up and those with prevalent CMDs at baseline, further supporting the robustness of the results. Fourth, although multivariable adjustment was performed, residual confounding from unmeasured confounders or measurement errors cannot be fully excluded (such as diet and physical activity). Nevertheless, E-value analyses show that only exceptionally potent unmeasured confounders could alter these results, implying limited residual confounding and supporting the robustness of our core findings. Moreover, the multiplicative FI-CHG model may amplify the influence of extreme values. In addition, lipid-lowering and glucose-lowering medications may directly affect TC, HDL-C, and FBG levels, potentially altering CHG values and introducing measurement-related bias into FI-CHG estimation. However, sensitivity analyses excluding participants using these medications yielded consistent results, supporting the reliability of the findings. Finally, this study included only Chinese adults aged ≥45 years. The clinical utility of these results needs further validation across diverse ethnicities, age groups and lifestyles. Future research should verify our findings in independent cohorts and varied populations to enhance generalizability.

## 5 Conclusion

In this cohort study, baseline FI-CHG levels, cumulative exposure, and longitudinal trajectory patterns were each significantly associated with incident cardiometabolic multimorbidity. These relationships exhibited a clear dose–response pattern and remained significant after adjusting for sociodemographic, behavioral, and clinical confounders. Overall, these findings support FI-CHG as a potential integrative biomarker for refining cardiovascular risk stratification. By integrating metabolic and functional indicators, the FI-CHG index may serve as a relatively robust metric with potential clinical prospects for risk stratification of chronic multimorbidity. It may help improve early risk identification and provide evidence for preventive strategies targeting cardiometabolic health.

## Data Availability

Publicly available datasets were analyzed in this study. This data can be found at: https://charls.pku.edu.cn/.

## Glossary

AUC: Area under the curve
BMI: Body mass index
CAD: Coronary artery disease
CHARLS: China health and retirement longitudinal study
CHG: Cholesterol, high-density lipoprotein, and glucose index
CI: Confidence interval
CMM: Cardiometabolic multimorbidity
CMD: Cardiometabolic diseases
CRP: C-reactive protein
CVD: Cardiovascular disease
DM: Diabetes mellitus
eGFR: Estimated glomerular filtration rate
FBG: Fasting blood glucose
FI: Frailty index
HbA1c: Hemoglobin A1c
HR: Hazard Ratio
IDI: Integrated discrimination improvement
IR: Insulin resistance
LDL-C: Low-density lipoprotein cholesterol
NGR: Normal glucose regulation
NRI: Net reclassification improvement
RCS: Restricted cubic spline
SD: Standard deviation
TC: Total cholesterol
T2D: Type 2 diabetes mellitus
TyG: Triglyceride-glucose index
UA: Uric acid
WSS: Within-cluster sum of squares
