# Supplementary material for: Combined associations of the frailty index and CHG index with cardiometabolic multimorbidity: a longitudinal study from the CHARLS cohort
Source: Front Nutr. 2026 Jun 5;13:1867031. doi: 10.3389/fnut.2026.1867031 (PMC13278977; doi:10.3389/fnut.2026.1867031)
Supplement: Supplementary file 1 [file Table_1.docx]

**Supplementary materials**

**Combined Associations of the Frailty Index and CHG Index with Cardiometabolic Multimorbidity: A Longitudinal Study from the CHARLS Cohort**

Lin Huang^1#^, Xuefang Yan^1#^, Youqin Wang^1^, Yun Ti^1^, Peili Bu^1*^, Jingyuan Li^1*^

1 State Key Laboratory for Innovation and Transformation of Luobing Theory; Key Laboratory of Cardiovascular Remodeling and Function Research of MOE, NHC, CAMS and Shandong Province; Department of Cardiology, Qilu Hospital of Shandong University, Jinan, China

**# These authors contributed equally to this work**

*** Corresponding author: Jingyuan Li** ([lijingyuan@sdu.edu.cn)](mailto:(lijingyuan@email.sdu.edu.cn)), **Peili Bu** [(bupeili@outlook.com](mailto:(bupeili@outlook.com;))

**Figure legends**

**Table S1.** Baseline characteristics of participants stratified by CMM status.

**Table S2.** Baseline characteristics of participants stratified by FI-CHG changes.

**Table S3.** Baseline characteristics of participants according to the cumulative FI-CHG tertiles.

**Table S4.** Incremental predictive value of FI-CHG compared with FI and CHG based on cNRI and IDI analyses.

**Table S5.** Sensitivity Analysis of association between baseline or cumulative FI-CHG and CMM risk excluding participants with any missing data.

**Table S6.** Sensitivity Analysis of association between baseline or cumulative FI-CHG and CMM risk excluding participants with any chronic disease (hypertension, diabetes, heart disease or stroke) at baseline.

**Table S7.** Sensitivity Analysis of association between baseline or cumulative FI-CHG and CMM risk excluding participants receiving lipid-lowering or glucose-lowering therapy at baseline.

**Table S8.** Sensitivity analysis of association between baseline or cumulative FI-CHG and CMM risk excluding participants who developed CMM within the first two years of follow-up.

**Table S9.** Associations of FI-CHG indicators with incident CMM and corresponding E‑values for assessing unmeasured confounding.

**Table S10.** Sensitivity analysis of the association between two-cluster FI-CHG trajectory groups and CMM risk.

**Figure S1.** Classification of change in the FI-CHG index from 2012 to 2015.

**Figure S2.** Comparison of the predictive value of FI, CHG and FI-CHG for CMM risk.

**Figure S3.** Age-associated risk of baseline FI-CHG for CMM incidence.

**Figure S4.** Age-associated risk of cumFI-CHG for CMM incidence.

**Table S1.** Baseline characteristics of participants stratified by CMM status.

| **Characteristics** | **Overall (8543)** | **Non-CMM(6877)** | **CMM(1666)** | ***P*** |
| --- | --- | --- | --- | --- |
| **Age** | 57.0 (51.0, 65.0) | 57.0 (51.0, 64.0) | 59.0 (54.0, 66.0) | <0.001 |
| **Gender** |  |  |  | <0.001 |
| Female | 4507 (52.8) | 3551 (51.6) | 956 (57.4) |  |
| Male | 4036 (47.2) | 3326 (48.4) | 710 (42.6) |  |
| **Education level** |  |  |  | <0.001 |
| Illiterate | 927 (10.9) | 768 (11.2) | 159 (9.5) |  |
| Sishu/homeschool  /elementary school | 2483 (29.1) | 1934 (28.1) | 549 (33.0) |  |
| Middle school | 1730 (20.3) | 1426 (20.7) | 304 (18.2) |  |
| High school and above | 3403 (39.8) | 2749 (40.0) | 654 (39.3) |  |
| **Marital status** |  |  |  | 0.005 |
| Non-married | 7566 (88.6) | 6124 (89.1) | 1442 (86.6) |  |
| Married | 977 (11.4) | 753 (10.9) | 224 (13.4) |  |
| **Location** |  |  |  | 0.265 |
| Village | 7053 (82.6) | 5693 (82.8) | 1360 (81.6) |  |
| City/town | 1490 (17.4) | 1184 (17.2) | 306 (18.4) |  |
| **Smoking status** |  |  |  | <0.001 |
| No | 5178 (60.6) | 4101 (59.6) | 1077 (64.6) |  |
| Yes | 3365 (39.4) | 2776 (40.4) | 589 (35.4) |  |
| **Drinking status** |  |  |  | 0.230 |
| No | 5149 (60.3) | 4123 (60.0) | 1026 (61.6) |  |
| Yes | 3394 (39.7) | 2754 (40.0) | 640 (38.4) |  |
| **SBP (mmHg)** | 126.0 (113.7, 141.3) | 124.0 (112.3, 138.7) | 134.3 (122.1, 149.0) | <0.001 |
| **DBP (mmHg)** | 74.3 (66.7, 83.3) | 73.7 (66.0, 82.3) | 78.3 (69.7, 86.7) | <0.001 |
| **BMI (Kg/m2)** | 23.1 (20.8, 25.7) | 22.7 (20.5, 25.3) | 24.6 (22.0, 27.4) | <0.001 |
| **FBG(mg/dl)** | 101.7 (94.1, 111.2) | 101.0 (93.8, 109.6) | 105.5 (96.5, 119.0) | <0.001 |
| **HbA1c (%)** | 5.1 (4.9, 5.4) | 5.1 (4.9, 5.4) | 5.2 (5.0, 5.6) | <0.001 |
| **UA(mg/dl)** | 4.3 (3.6, 5.1) | 4.3 (3.6, 5.1) | 4.3 (3.6, 5.2) | 0.110 |
| **eGFR(mL/min/1.73 m2)** | 95.0 (84.6, 102.5) | 95.3 (85.1, 103.0) | 93.6 (83.5, 100.6) | <0.001 |
| **CRP (mg/dl)** | 1.0 (0.5, 2.1) | 0.9 (0.5, 1.9) | 1.2 (0.6, 2.6) | <0.001 |
| **TG (mg/dl)** | 102.7 (73.5, 148.7) | 99.1 (71.7, 143.4) | 118.6 (83.2, 170.8) | <0.001 |
| **TC (mg/dl)** | 191.0 (167.4, 215.7) | 189.8 (167.0, 214.9) | 194.1 (170.1, 220.0) | <0.001 |
| **LDL (mg/dl)** | 115.2 (94.3, 137.8) | 114.8 (93.9, 136.9) | 116.8 (95.9, 141.5) | 0.001 |
| **HDL (mg/dl)** | 49.9 (41.0, 60.3) | 50.6 (41.8, 61.5) | 47.2 (38.3, 57.2) | <0.001 |
| **FI** | 0.1 (0.1, 0.2) | 0.1 (0.1, 0.2) | 0.1 (0.1, 0.2) | <0.001 |
| **CHG** | 5.3 (5.0, 5.5) | 5.2 (5.0, 5.5) | 5.4 (5.1, 5.7) | <0.001 |
| **FI-CHG** | 0.5 (0.3, 1.0) | 0.5 (0.3, 0.9) | 0.7 (0.4, 1.2) | <0.001 |
| **Hypertension** |  |  |  | <0.001 |
| No | 6859 (80.3) | 5845 (85.0) | 1014 (60.9) |  |
| Yes | 1684 (19.7) | 1032 (15.0) | 652 (39.1) |  |
| **Diabetes** |  |  |  | <0.001 |
| No | 8099 (94.8) | 6634 (96.5) | 1465 (87.9) |  |
| Yes | 444 (5.2) | 243 (3.5) | 201 (12.1) |  |
| **Heart disease** |  |  |  | <0.001 |
| No | 8031 (94.0) | 6561 (95.4) | 1470 (88.2) |  |
| Yes | 512 (6.0) | 316 (4.6) | 196 (11.8) |  |
| **Stroke** |  |  |  | 0.001 |
| No | 8477 (99.2) | 6835 (99.4) | 1642 (98.6) |  |
| Yes | 66 (0.8) | 42 (0.6) | 24 (1.4) |  |
| **Dyslipidemia** |  |  |  | <0.001 |
| No | 7862 (92.0) | 6440 (93.6) | 1422 (85.4) |  |
| Yes | 681 (8.0) | 437 (6.4) | 244 (14.6) |  |
| **Antihypertensive drugs** |  |  |  | <0.001 |
| No | 7327 (85.8) | 6175 (89.8) | 1152 (69.1) |  |
| Yes | 1216 (14.2) | 702 (10.2) | 514 (30.9) |  |
| **Hypoglycemic drugs** |  |  |  | <0.001 |
| No | 8392 (98.2) | 6798 (98.9) | 1594 (95.7) |  |
| Yes | 151 (1.8) | 79 (1.1) | 72 (4.3) |  |
| **Lipid-lowering drugs** |  |  |  | <0.001 |
| No | 8232 (96.4) | 6683 (97.2) | 1549 (93.0) |  |
| Yes | 311 (3.6) | 194 (2.8) | 117 (7.0) |  |

Abbreviations: CMM, cardiometabolic multimorbidity; SBP, systolic blood pressure; DBP, diastolic blood pressure; BMI, body mass index; FBG, fasting blood glucose; HbA1c, hemoglobin A1c; UA, uric acid; eGFR, estimated glomerular filtration rate; CRP, C-reactive protein; TG, triglycerides; TC, total cholesterol; LDL, low-density Lipoprotein cholesterol; HDL, high-density lipoprotein cholesterol; FI, frailty index; CHG, Cholesterol, High-Density Lipoprotein, Glucose index.

**Table S2.** Baseline characteristics of participants stratified by FI-CHG changes.

| **Characteristics** | **Overall (5750)** | **Cluster 1 (3366)** | **Cluster 2 (1854)** | **Cluster 3 (530)** | ***P*** |
| --- | --- | --- | --- | --- | --- |
| **Age** | 58.0 (52.0, 64.0) | 56.0 (50.0, 62.0) | 59.0 (53.0, 65.0) | 63.0 (57.0, 69.0) | <0.001 |
| **Gender** |  |  |  |  | <0.001 |
| Female | 3077 (53.5) | 1586 (47.1) | 1143 (61.7) | 348 (65.7) |  |
| Male | 2673 (46.5) | 1780 (52.9) | 711 (38.3) | 182 (34.3) |  |
| **Education level** |  |  |  |  | <0.001 |
| Illiterate | 1673 (29.1) | 769 (22.8) | 672 (36.2) | 232 (43.8) |  |
| Sishu/homeschool  /elementary school | 2377 (41.3) | 1351 (40.1) | 789 (42.6) | 237 (44.7) |  |
| Middle school | 1158 (20.1) | 817 (24.3) | 295 (15.9) | 46 (8.7) |  |
| High school and above | 542 (9.4) | 429 (12.7) | 98 (5.3) | 15 (2.8) |  |
| **Marital status** |  |  |  |  | <0.001 |
| Non-married | 600 (10.4) | 255 (7.6) | 261 (14.1) | 84 (15.8) |  |
| Married | 5150 (89.6) | 3111 (92.4) | 1593 (85.9) | 446 (84.2) |  |
| **Location** |  |  |  |  | <0.001 |
| Village | 4881 (84.9) | 2762 (82.1) | 1640 (88.5) | 479 (90.4) |  |
| City/town | 869 (15.1) | 604 (17.9) | 214 (11.5) | 51 (9.6) |  |
| **Smoking status** |  |  |  |  | <0.001 |
| No | 3503 (60.9) | 1926 (57.2) | 1226 (66.1) | 351 (66.2) |  |
| Yes | 2247 (39.1) | 1440 (42.8) | 628 (33.9) | 179 (33.8) |  |
| **Drinking status** |  |  |  |  | 0.001 |
| No | 3467 (60.3) | 1961 (58.3) | 1167 (62.9) | 339 (64.0) |  |
| Yes | 2283 (39.7) | 1405 (41.7) | 687 (37.1) | 191 (36.0) |  |
| **SBP (mmHg)** | 125.3 (113.3, 140.0) | 125.0 (113.7, 139.3) | 125.7 (112.7, 140.0) | 128.7 (115.0, 143.9) | 0.002 |
| **DBP (mmHg)** | 74.3 (66.7, 82.7) | 74.7 (67.0, 83.0) | 73.7 (66.0, 82.3) | 74.3 (66.7, 83.0) | 0.020 |
| **BMI (Kg/m2)** | 23.0 (20.8, 25.6) | 23.1 (21.0, 25.4) | 23.0 (20.6, 25.9) | 23.1 (20.8, 26.0) | 0.598 |
| **FBG(mg/dl)** | 101.7 (94.3, 110.7) | 101.5 (94.3, 110.2) | 101.5 (94.1, 110.7) | 103.0 (94.9, 113.7) | 0.036 |
| **HbA1c (%)** | 5.1 (4.9, 5.4) | 5.1 (4.9, 5.4) | 5.2 (4.9, 5.5) | 5.2 (4.9, 5.5) | <0.001 |
| **UA(mg/dl)** | 4.2 (3.5, 5.1) | 4.3 (3.6, 5.1) | 4.1 (3.5, 5.0) | 4.2 (3.4, 4.9) | <0.001 |
| **eGFR(mL/min/1.73 m2)** | 95.6 (85.8, 102.6) | 96.4 (86.8, 103.6) | 95.2 (85.2, 102.0) | 92.0 (81.8, 99.1) | <0.001 |
| **CRP (mg/dl)** | 1.0 (0.5, 2.0) | 0.9 (0.5, 1.8) | 1.0 (0.6, 2.2) | 1.2 (0.6, 2.5) | <0.001 |
| **TG (mg/dl)** | 101.8 (73.5, 148.7) | 97.3 (71.7, 146.0) | 106.2 (74.3, 153.1) | 109.7 (80.5, 156.4) | <0.001 |
| **TC (mg/dl)** | 191.0 (167.4, 215.3) | 189.4 (166.2, 213.4) | 192.3 (168.9, 218.8) | 195.2 (169.0, 219.6) | <0.001 |
| **LDL (mg/dl)** | 114.8 (94.3, 137.2) | 114.4 (94.3, 135.7) | 115.2 (93.9, 139.2) | 117.9 (94.8, 140.2) | 0.076 |
| **HDL (mg/dl)** | 49.9 (41.0, 60.3) | 49.9 (40.6, 60.3) | 50.3 (41.0, 60.6) | 49.5 (40.2, 59.5) | 0.594 |
| **FI** | 0.1 (0.1, 0.2) | 0.1 (0.0, 0.1) | 0.2 (0.1, 0.2) | 0.4 (0.3, 0.5) | <0.001 |
| **CHG** | 5.3 (5.0, 5.5) | 5.2 (5.0, 5.5) | 5.3 (5.0, 5.5) | 5.3 (5.1, 5.6) | 0.002 |
| **FI-CHG** | 0.5 (0.3, 0.9) | 0.3 (0.2, 0.5) | 0.9 (0.7, 1.2) | 1.9 (1.5, 2.4) | <0.001 |
| **Hypertension** |  |  |  |  | <0.001 |
| No | 4603 (80.1) | 2799 (83.2) | 1427 (77.0) | 377 (71.1) |  |
| Yes | 1147 (19.9) | 567 (16.8) | 427 (23.0) | 153 (28.9) |  |
| **Diabetes** |  |  |  |  | 0.024 |
| No | 5456 (94.9) | 3215 (95.5) | 1747 (94.2) | 494 (93.2) |  |
| Yes | 294 (5.1) | 151 (4.5) | 107 (5.8) | 36 (6.8) |  |
| **Heart disease** |  |  |  |  | <0.001 |
| No | 5404 (94.0) | 3237 (96.2) | 1716 (92.6) | 451 (85.1) |  |
| Yes | 346 (6.0) | 129 (3.8) | 138 (7.4) | 79 (14.9) |  |
| **Stroke** |  |  |  |  | <0.001 |
| No | 5704 (99.2) | 3352 (99.6) | 1834 (98.9) | 518 (97.7) |  |
| Yes | 46 (0.8) | 14 (0.4) | 20 (1.1) | 12 (2.3) |  |
| **Dyslipidemia** |  |  |  |  | <0.001 |
| No | 5270 (91.7) | 3124 (92.8) | 1675 (90.3) | 471 (88.9) |  |
| Yes | 480 (8.3) | 242 (7.2) | 179 (9.7) | 59 (11.1) |  |
| **Antihypertensive drugs** |  |  |  |  | <0.001 |
| No | 4937 (85.9) | 2981 (88.6) | 1543 (83.2) | 413 (77.9) |  |
| Yes | 813 (14.1) | 385 (11.4) | 311 (16.8) | 117 (22.1) |  |
| **Hypoglycemic drugs** |  |  |  |  | 0.100 |
| No | 5649 (98.2) | 3317 (98.5) | 1812 (97.7) | 520 (98.1) |  |
| Yes | 101 (1.8) | 49 (1.5) | 42 (2.3) | 10 (1.9) |  |
| **Lipid-lowering drugs** |  |  |  |  | <0.001 |
| No | 5523 (96.1) | 3263 (96.9) | 1764 (95.1) | 496 (93.6) |  |
| Yes | 227 (3.9) | 103 (3.1) | 90 (4.9) | 34 (6.4) |  |
| **CMM outcome** |  |  |  |  | <0.001 |
| No | 4523 (78.7) | 2869 (85.2) | 1343 (72.4) | 311 (58.7) |  |
| Yes | 1227 (21.3) | 497 (14.8) | 511 (27.6) | 219 (41.3) |  |

Abbreviations: FI, frailty index; CHG, Cholesterol, High-Density Lipoprotein, Glucose index; SBP, systolic blood pressure; DBP, diastolic blood pressure; BMI, body mass index; FBG, fasting blood glucose; HbA1c, hemoglobin A1c; UA, uric acid; eGFR, estimated glomerular filtration rate; CRP, C-reactive protein; TG, triglycerides; TC, total cholesterol; LDL, low-density Lipoprotein cholesterol; HDL, high-density lipoprotein cholesterol; CMM, cardiometabolic multimorbidity.

**Table S3.** Baseline characteristics of participants according to the cumulative FI-CHG tertiles.

| **Characteristics** | **Overall (5750)** | **T1 (1917)** | **T2 (1916)** | **T3 (1917)** | ***P*** |
| --- | --- | --- | --- | --- | --- |
| **Age** | 58.0 (52.0, 64.0) | 56.0 (49.0, 61.0) | 57.0 (51.0, 64.0) | 60.0 (54.0, 67.0) | <0.001 |
| **Gender** |  |  |  |  | <0.001 |
| Female | 3077 (53.5) | 828 (43.2) | 1034 (54.0) | 1215 (63.4) |  |
| Male | 2673 (46.5) | 1089 (56.8) | 882 (46.0) | 702 (36.6) |  |
| **Education level** |  |  |  |  | <0.001 |
| Illiterate | 1673 (29.1) | 396 (20.7) | 509 (26.6) | 768 (40.1) |  |
| Sishu/homeschool  /elementary school | 2377 (41.3) | 725 (37.8) | 836 (43.6) | 816 (42.6) |  |
| Middle school | 1158 (20.1) | 509 (26.6) | 399 (20.8) | 250 (13.0) |  |
| High school and above | 542 (9.4) | 287 (15.0) | 172 (9.0) | 83 (4.3) |  |
| **Marital status** |  |  |  |  | <0.001 |
| Non-married | 600 (10.4) | 123 (6.4) | 184 (9.6) | 293 (15.3) |  |
| Married | 5150 (89.6) | 1794 (93.6) | 1732 (90.4) | 1624 (84.7) |  |
| **Location** |  |  |  |  | <0.001 |
| Village | 4881 (84.9) | 1544 (80.5) | 1621 (84.6) | 1716 (89.5) |  |
| City/town | 869 (15.1) | 373 (19.5) | 295 (15.4) | 201 (10.5) |  |
| **Smoking status** |  |  |  |  | <0.001 |
| No | 3503 (60.9) | 1076 (56.1) | 1157 (60.4) | 1270 (66.2) |  |
| Yes | 2247 (39.1) | 841 (43.9) | 759 (39.6) | 647 (33.8) |  |
| **Drinking status** |  |  |  |  | <0.001 |
| No | 3467 (60.3) | 1078 (56.2) | 1170 (61.1) | 1219 (63.6) |  |
| Yes | 2283 (39.7) | 839 (43.8) | 746 (38.9) | 698 (36.4) |  |
| **SBP (mmHg)** | 125.3 (113.3, 140.0) | 124.7 (113.7, 139.0) | 125.3 (113.3, 140.1) | 126.7 (113.3, 141.0) | 0.177 |
| **DBP (mmHg)** | 74.3 (66.7, 82.7) | 74.3 (67.0, 83.3) | 74.3 (66.7, 83.0) | 73.7 (66.3, 82.7) | 0.083 |
| **BMI (Kg/m2)** | 23.0 (20.8, 25.6) | 23.1 (21.1, 25.4) | 23.0 (20.8, 25.6) | 23.0 (20.6, 25.9) | 0.489 |
| **FBG(mg/dl)** | 101.7 (94.3, 110.7) | 101.5 (94.3, 110.0) | 101.7 (94.3, 110.5) | 101.7 (94.3, 111.8) | 0.440 |
| **HbA1c (%)** | 5.1 (4.9, 5.4) | 5.1 (4.8, 5.4) | 5.1 (4.9, 5.4) | 5.2 (4.9, 5.5) | <0.001 |
| **UA(mg/dl)** | 4.2 (3.5, 5.1) | 4.3 (3.6, 5.2) | 4.2 (3.5, 5.1) | 4.1 (3.5, 4.9) | <0.001 |
| **eGFR(mL/min/1.73 m2)** | 95.6 (85.8, 102.6) | 96.8 (87.4, 104.3) | 95.9 (85.0, 102.5) | 94.4 (84.5, 101.3) | <0.001 |
| **CRP (mg/dl)** | 1.0 (0.5, 2.0) | 0.9 (0.5, 1.8) | 0.9 (0.5, 1.9) | 1.1 (0.6, 2.4) | <0.001 |
| **TG (mg/dl)** | 101.8 (73.5, 148.7) | 96.5 (70.8, 146.0) | 101.8 (74.3, 147.8) | 106.2 (75.2, 152.2) | <0.001 |
| **TC (mg/dl)** | 191.0 (167.4, 215.3) | 187.9 (165.5, 211.5) | 191.9 (167.8, 215.7) | 192.9 (168.9, 219.2) | <0.001 |
| **LDL (mg/dl)** | 114.8 (94.3, 137.2) | 113.3 (93.2, 134.5) | 115.6 (94.3, 138.8) | 116.4 (94.7, 139.6) | 0.005 |
| **HDL (mg/dl)** | 49.9 (41.0, 60.3) | 49.9 (41.0, 61.1) | 49.9 (40.6, 59.5) | 49.9 (41.0, 60.7) | 0.475 |
| **FI** | 0.1 (0.1, 0.2) | 0.0 (0.0, 0.1) | 0.1 (0.1, 0.1) | 0.2 (0.2, 0.3) | <0.001 |
| **CHG** | 5.3 (5.0, 5.5) | 5.2 (5.0, 5.5) | 5.3 (5.0, 5.5) | 5.3 (5.0, 5.5) | 0.001 |
| **FI-CHG** | 0.5 (0.3, 0.9) | 0.2 (0.1, 0.3) | 0.5 (0.4, 0.7) | 1.2 (0.8, 1.6) | <0.001 |
| **cumFI-CHG** |  |  |  |  | <0.001 |
| **Hypertension** | 4603 (80.1) | 1630 (85.0) | 1523 (79.5) | 1450 (75.6) |  |
| No | 1147 (19.9) | 287 (15.0) | 393 (20.5) | 467 (24.4) |  |
| Yes |  |  |  |  | 0.056 |
| **Diabetes** | 5456 (94.9) | 1837 (95.8) | 1814 (94.7) | 1805 (94.2) |  |
| No | 294 (5.1) | 80 (4.2) | 102 (5.3) | 112 (5.8) |  |
| Yes |  |  |  |  | <0.001 |
| **Heart disease** | 5404 (94.0) | 1845 (96.2) | 1833 (95.7) | 1726 (90.0) |  |
| No | 346 (6.0) | 72 (3.8) | 83 (4.3) | 191 (10.0) |  |
| Yes |  |  |  |  | <0.001 |
| **Stroke** | 5704 (99.2) | 1912 (99.7) | 1902 (99.3) | 1890 (98.6) |  |
| No | 46 (0.8) | 5 (0.3) | 14 (0.7) | 27 (1.4) |  |
| Yes |  |  |  |  | <0.001 |
| **Dyslipidemia** | 5270 (91.7) | 1794 (93.6) | 1753 (91.5) | 1723 (89.9) |  |
| No | 480 (8.3) | 123 (6.4) | 163 (8.5) | 194 (10.1) |  |
| Yes |  |  |  |  | <0.001 |
| **Antihypertensive drugs** | 4937 (85.9) | 1733 (90.4) | 1633 (85.2) | 1571 (82.0) |  |
| No | 813 (14.1) | 184 (9.6) | 283 (14.8) | 346 (18.0) |  |
| Yes |  |  |  |  | 0.218 |
| **Hypoglycemic drugs** | 5649 (98.2) | 1891 (98.6) | 1881 (98.2) | 1877 (97.9) |  |
| No | 101 (1.8) | 26 (1.4) | 35 (1.8) | 40 (2.1) |  |
| Yes |  |  |  |  | <0.001 |
| **Lipid-lowering drugs** | 5523 (96.1) | 1864 (97.2) | 1849 (96.5) | 1810 (94.4) |  |
| No | 227 (3.9) | 53 (2.8) | 67 (3.5) | 107 (5.6) |  |
| Yes |  |  |  |  | <0.001 |
| **CMM outcome** | 4523 (78.7) | 1692 (88.3) | 1540 (80.4) | 1291 (67.3) |  |
| No | 1227 (21.3) | 225 (11.7) | 376 (19.6) | 626 (32.7) |  |
| Yes | 58.0 (52.0, 64.0) | 56.0 (49.0, 61.0) | 57.0 (51.0, 64.0) | 60.0 (54.0, 67.0) | <0.001 |

Abbreviations: FI, frailty index; CHG, Cholesterol, High-Density Lipoprotein, Glucose index; SBP, systolic blood pressure; DBP, diastolic blood pressure; BMI, body mass index; FBG, fasting blood glucose; HbA1c, hemoglobin A1c; UA, uric acid; eGFR, estimated glomerular filtration rate; CRP, C-reactive protein; TG, triglycerides; TC, total cholesterol; LDL, low-density Lipoprotein cholesterol; HDL, high-density lipoprotein cholesterol; cumFI-CHG, cumulative FI-CHG; CMM, cardiometabolic multimorbidity.

**Table S4.** Incremental predictive value of FI-CHG compared with FI and CHG based on cNRI and IDI analyses.

| **Variables** | **Net Reclassification Improvement** | | **Integrated Discrimination Improvement** | |
| --- | --- | --- | --- | --- |
|  | **cNRI (95%CI)** | ***P*** | **IDI (95%CI)** | ***P*** |
| FI | 0.152 (0.072-0.214) | < 0.001 | 0.005 (0.001-0.009) | 0.004 |
| CHG | 0.073 (0.007-0.140) | 0.034 | 0.002 (-0.002-0.006) | 0.248 |
| FI-CHG | 0.148 (0.083-0.223) | < 0.001 | 0.006 (0.001-0.011) | 0.004 |

Model: Age, gender, location, education, marital, smoking, drinking, dyslipidemia, CRP, UA, and HbA1c were adjusted.

Abbreviations: FI, frailty index; CHG, Cholesterol, High-Density Lipoprotein, Glucose index; cNRI, continuous net reclassification improvement; IDI, integrated discrimination improvement; CI, confidence interval.

**Table S5.** Sensitivity Analysis of association between baseline or cumulative FI-CHG and CMM risk excluding participants with any missing data.

| **Variables** | **Crude model** | | **Model 1** | | **Model 2** | |
| --- | --- | --- | --- | --- | --- | --- |
|  | **HR (95%CI)** | ***P*** | **HR (95%CI)** | ***P*** | **HR (95%CI)** | ***P*** |
| **FI-CHG** |  |  |  |  |  |  |
| Per 1 unit | 1.650 (1.541-1.768) | <0.001 | 1.569 (1.456-1.689) | <0.001 | 1.511 (1.401-1.628) | <0.001 |
| Quartile |  |  |  |  |  |  |
| Q1 | Ref |  | Ref |  | Ref |  |
| Q2 | 1.222 (1.026-1.457) | 0.025 | 1.207 (1.012-1.438) | 0.036 | 1.183 (0.993-1.411) | 0.061 |
| Q3 | 1.643 (1.392-1.940) | <0.001 | 1.597 (1.351-1.888) | <0.001 | 1.553 (1.313-1.835) | <0.001 |
| Q4 | 2.579 (2.207-3.012) | <0.001 | 2.390 (2.033-2.810) | <0.001 | 2.221 (1.888-2.613) | <0.001 |
| P for trend |  | <0.001 |  | <0.001 |  | <0.001 |
| **cumFI-CHG** |  |  |  |  |  |  |
| Per 1 unit | 1.259 (1.225-1.294) | <0.001 | 1.241 (1.205-1.278) | <0.001 | 1.230 (1.193-1.267) | <0.001 |
| Tertile |  |  |  |  |  |  |
| T1 | Ref |  | Ref |  | Ref |  |
| T2 | 1.863 (1.554-2.234) | <0.001 | 1.825 (1.520-2.192) | <0.001 | 1.761 (1.466-2.115) | <0.001 |
| T3 | 3.376 (2.854-3.993) | <0.001 | 3.182 (2.669-3.793) | <0.001 | 2.994 (2.510-3.571) | <0.001 |
| P for trend |  | <0.001 |  | <0.001 |  | <0.001 |

Crude model: No covariates were adjusted; Model 1: Age, gender, location, education, marital, smoking and drinking status were adjusted; Model 2: dyslipidemia, CRP, UA, and HbA1c were further adjusted.

Abbreviations: FI, frailty index; CHG, Cholesterol, High-Density Lipoprotein, Glucose index; CMM, cardiometabolic multimorbidity; CRP, C-reactive protein; UA, uric acid; HbA1c, hemoglobin A1c; HR, Hazard Ratio; CI, confidence interval.

**Table S6.** Sensitivity Analysis of association between baseline or cumulative FI-CHG and CMM risk excluding participants with any chronic disease (hypertension, diabetes, heart disease or stroke) at baseline.

| **Variables** | **Crude model** | | **Model 1** | | **Model 2** | |
| --- | --- | --- | --- | --- | --- | --- |
|  | **HR (95%CI)** | ***P*** | **HR (95%CI)** | ***P*** | **HR (95%CI)** | ***P*** |
| **FI-CHG** |  |  |  |  |  |  |
| Per 1 unit | 1.671 (1.510-1.849) | <0.001 | 1.587 (1.419-1.774) | <0.001 | 1.533 (1.369-1.715) | <0.001 |
| Quartile |  |  |  |  |  |  |
| Q1 | Ref |  | Ref |  | Ref |  |
| Q2 | 1.503 (1.149-1.966) | 0.003 | 1.485 (1.134-1.944) | 0.004 | 1.467 (1.121-1.921) | 0.005 |
| Q3 | 1.843 (1.422-2.388) | <0.001 | 1.806 (1.392-2.345) | <0.001 | 1.790 (1.379-2.324) | <0.001 |
| Q4 | 3.015 (2.367-3.839) | <0.001 | 2.798 (2.177-3.597) | <0.001 | 2.637 (2.050-3.393) | <0.001 |
| P for trend |  | <0.001 |  | <0.001 |  | <0.001 |
| **cumFI-CHG** |  |  |  |  |  |  |
| Per 1 unit | 1.272 (1.219-1.327) | <0.001 | 1.256 (1.199-1.315) | <0.001 | 1.246 (1.189-1.306) | <0.001 |
| Tertile |  |  |  |  |  |  |
| T1 | Ref |  | Ref |  | Ref |  |
| T2 | 2.020 (1.530-2.668) | <0.001 | 1.984 (1.500-2.623) | <0.001 | 1.946 (1.471-2.574) | <0.001 |
| T3 | 3.558 (2.748-4.606) | <0.001 | 3.375 (2.581-4.412) | <0.001 | 3.210 (2.453-4.199) | <0.001 |
| P for trend |  | <0.001 |  | <0.001 |  | <0.001 |

Crude model: No covariates were adjusted; Model 1: Age, gender, location, education, marital, smoking and drinking status were adjusted; Model 2: dyslipidemia, CRP, UA, and HbA1c were further adjusted.

Abbreviations: FI, frailty index; CHG, Cholesterol, High-Density Lipoprotein, Glucose index; CMM, cardiometabolic multimorbidity; CRP, C-reactive protein; UA, uric acid; HbA1c, hemoglobin A1c; HR, Hazard Ratio; CI, confidence interval.

**Table S7.** Sensitivity Analysis of association between baseline or cumulative FI-CHG and CMM risk excluding participants receiving lipid-lowering or glucose-lowering therapy at baseline.

| **Variables** | **Crude model** | | **Model 1** | | **Model 2** | |
| --- | --- | --- | --- | --- | --- | --- |
|  | **HR (95%CI)** | **P** | **HR (95%CI)** | **P** | **HR (95%CI)** | **P** |
| **FI-CHG** |  |  |  |  |  |  |
| Per 1 unit | 1.583 (1.489-1.682) | <0.001 | 1.514 (1.417-1.618) | <0.001 | 1.452 (1.359-1.552) | <0.001 |
| Quartile |  |  |  |  |  |  |
| Q1 | Ref |  | Ref |  | Ref |  |
| Q2 | 1.281 (1.091-1.504) | 0.002 | 1.266 (1.078-1.486) | 0.004 | 1.240 (1.056-1.456) | 0.009 |
| Q3 | 1.638 (1.405-1.909) | <0.001 | 1.596 (1.368-1.863) | <0.001 | 1.557 (1.334-1.818) | <0.001 |
| Q4 | 2.521 (2.184-2.911) | <0.001 | 2.355 (2.027-2.736) | <0.001 | 2.204 (1.896-2.563) | <0.001 |
| P for trend |  | <0.001 |  | <0.001 |  | <0.001 |
| **cumFI-CHG** |  |  |  |  |  |  |
| Per 1 unit | 1.255 (1.224-1.287) | <0.001 | 1.239 (1.206-1.273) | <0.001 | 1.226 (1.193-1.260) | <0.001 |
| Tertile |  |  |  |  |  |  |
| T1 | Ref |  | Ref |  | Ref |  |
| T2 | 1.730 (1.465-2.044) | <0.001 | 1.702 (1.438-2.013) | <0.001 | 1.640 (1.386-1.941) | <0.001 |
| T3 | 3.154 (2.705-3.678) | <0.001 | 2.996 (2.551-3.519) | <0.001 | 2.821 (2.401-3.315) | <0.001 |
| P for trend |  | <0.001 |  | <0.001 |  | <0.001 |

Crude model: No covariates were adjusted; Model 1: Age, gender, location, education, marital, smoking and drinking status were adjusted; Model 2: dyslipidemia, CRP, UA, and HbA1c were further adjusted.

Abbreviations: FI, frailty index; CHG, Cholesterol, High-Density Lipoprotein, Glucose index; CMM, cardiometabolic multimorbidity; CRP, C-reactive protein; UA, uric acid; HbA1c, hemoglobin A1c; HR, Hazard Ratio; CI, confidence interval.

**Table S8.** Sensitivity analysis of association between baseline or cumulative FI-CHG and CMM risk excluding participants who developed CMM within the first two years of follow-up.

| **Variables** | **Crude model** | | **Model 1** | | **Model 2** | |
| --- | --- | --- | --- | --- | --- | --- |
|  | **HR (95%CI)** | **P** | **HR (95%CI)** | **P** | **HR (95%CI)** | **P** |
| **FI-CHG** |  |  |  |  |  |  |
| Per 1 unit | 1.581 (1.481-1.687) | <0.001 | 1.511 (1.408-1.622) | <0.001 | 1.452 (1.353-1.559) | <0.001 |
| Quartile |  |  |  |  |  |  |
| Q1 | Ref |  | Ref |  | Ref |  |
| Q2 | 1.335 (1.128-1.580) | <0.001 | 1.319 (1.114-1.561) | 0.001 | 1.292 (1.092-1.530) | 0.003 |
| Q3 | 1.584 (1.345-1.865) | <0.001 | 1.538 (1.304-1.814) | <0.001 | 1.501 (1.273-1.770) | <0.001 |
| Q4 | 2.517 (2.161-2.932) | <0.001 | 2.337 (1.993-2.741) | <0.001 | 2.190 (1.866-2.570) | <0.001 |
| P for trend |  | <0.001 |  | <0.001 |  | <0.001 |
| **cumFI-CHG** |  |  |  |  |  |  |
| Per 1 unit | 1.257 (1.224-1.291) | <0.001 | 1.241 (1.206-1.277) | <0.001 | 1.228 (1.193-1.264) | <0.001 |
| Tertile |  |  |  |  |  |  |
| T1 | Ref |  | Ref |  | Ref |  |
| T2 | 1.699 (1.426-2.024) | <0.001 | 1.663 (1.394-1.985) | <0.001 | 1.601 (1.341-1.911) | <0.001 |
| T3 | 3.125 (2.660-3.672) | <0.001 | 2.942 (2.485-3.484) | <0.001 | 2.775 (2.342-3.288) | <0.001 |
| P for trend |  | <0.001 |  | <0.001 |  | <0.001 |

Crude model: No covariates were adjusted; Model 1: Age, gender, location, education, marital, smoking, and drinking were adjusted; Model 2: Dyslipidemia, CRP, UA, and HbA1c were further adjusted.

Abbreviations: FI, frailty index; CHG, Cholesterol, High-Density Lipoprotein, Glucose index; CMM, cardiometabolic multimorbidity; CRP, C-reactive protein; UA, uric acid; HbA1c, hemoglobin A1c; HR, Hazard Ratio; CI, confidence

**Table S9.** Associations of FI-CHG indicators with incident CMM and corresponding E‑values for assessing unmeasured confounding.

| **Variables** | **Crude model** | | | **Model 1** | | | **Model 2** |  |  |
| --- | --- | --- | --- | --- | --- | --- | --- | --- | --- |
|  | **HR (95%CI)** | **P** | **E value** | **HR (95%CI)** | **P** | **E value** | **HR (95%CI)** | **P** | **E value** |
| **FI-CHG** |  |  |  |  |  |  |  |  |  |
| Per 1 unit | 1.580 (1.488-1.678) | <0.001 | 2.540 | 1.515 (1.419-1.618) | <0.001 | 2.400 | 1.452 (1.360-1.551) | <0.001 | 2.260 |
| Quartile |  |  |  |  |  |  |  |  |  |
| Q1 | Ref |  |  | Ref |  |  | Ref |  |  |
| Q2 | 1.290 (1.101-1.512) | 0.002 | 1.902 | 1.275 (1.088-1.495) | 0.003 | 1.868 | 1.249 (1.065-1.464) | 0.006 | 1.806 |
| Q3 | 1.638 (1.407-1.907) | <0.001 | 2.660 | 1.598 (1.371-1.863) | <0.001 | 2.576 | 1.558 (1.337-1.816) | <0.001 | 2.491 |
| Q4 | 2.540 (2.203-2.930) | <0.001 | 4.519 | 2.382 (2.053-2.765) | <0.001 | 4.197 | 2.225 (1.917-2.584) | <0.001 | 3.877 |
| P for trend |  | <0.001 |  |  | <0.001 |  |  | <0.001 |  |
| **cumFI-CHG** |  |  |  |  |  |  |  |  |  |
| Per 1 unit | 1.255 (1.225-1.287) | <0.001 | 1.750 | 1.241 (1.208-1.275) | <0.001 | 1.710 | 1.228 (1.195-1.261) | <0.001 | 1.680 |
| Tertile |  |  |  |  |  |  |  |  |  |
| T1 | Ref |  |  | Ref |  |  | Ref |  |  |
| T2 | 1.758 (1.490-2.074) | <0.001 | 2.912 | 1.732 (1.466-2.046) | <0.001 | 2.857 | 1.664 (1.409-1.967) | <0.001 | 2.716 |
| T3 | 3.185 (2.735-3.710) | <0.001 | 5.823 | 3.041 (2.592-3.568) | <0.001 | 5.533 | 2.860 (2.436-3.357) | <0.001 | 5.166 |
| P for trend |  | <0.001 |  |  | <0.001 |  |  | <0.001 |  |
| **K-means Group** |  |  |  |  |  |  |  |  |  |
| Cluster1 | Ref |  |  | Ref |  |  | Ref |  |  |
| Cluster2 | 2.022 (1.787-2.288) | <0.001 | 3.460 | 1.961 (1.727-2.228) | <0.001 | 3.334 | 1.886 (1.661-2.143) | <0.001 | 3.179 |
| Cluster3 | 3.393 (2.894-3.979) | <0.001 | 6.243 | 3.151 (2.662-3.731) | <0.001 | 5.755 | 2.985 (2.521-3.534) | <0.001 | 5.418 |

Crude model: No covariates were adjusted; Model 1: Age, gender, location, education, marital, smoking, and drinking were adjusted; Model 2: Dyslipidemia, CRP, UA, and HbA1c were further adjusted.

Abbreviations: FI, frailty index; CHG, Cholesterol, High-Density Lipoprotein, Glucose index; CMM, cardiometabolic multimorbidity; CRP, C-reactive protein; UA, uric acid; HbA1c, hemoglobin A1c; HR, Hazard Ratio; CI, confidence

**Table S10.** Sensitivity analysis of the association between two-cluster FI-CHG trajectory groups and CMM risk.

| **Variables** | **Crude model** | | **Model 1** | | **Model 2** | |
| --- | --- | --- | --- | --- | --- | --- |
|  | **HR (95%CI)** | **P** | **HR (95%CI)** | **P** | **HR (95%CI)** | **P** |
| **Cluster** |  |  |  |  |  |  |
| Cluster 1 | Ref |  | Ref |  | Ref |  |
| Cluster 1 | 2.325 (2.074-2.607) | <0.001 | 2.173 (1.925-2.452) | <0.001 | 2.076 (1.840-2.343) | <0.001 |
| P for trend |  | <0.001 |  | <0.001 |  | <0.001 |

Crude model: No covariates were adjusted; Model 1: Age, gender, location, education, marital, smoking, and drinking were adjusted; Model 2: Dyslipidemia, CRP, UA, and HbA1c were further adjusted.

Abbreviations: FI, frailty index; CHG, Cholesterol, High-Density Lipoprotein, Glucose index; CMM, cardiometabolic multimorbidity; CRP, C-reactive protein; UA, uric acid; HbA1c, hemoglobin A1c; HR, Hazard Ratio; CI, confidence

**Figure S1.** Classification of change in the FI-CHG index from 2012 to 2015.

**
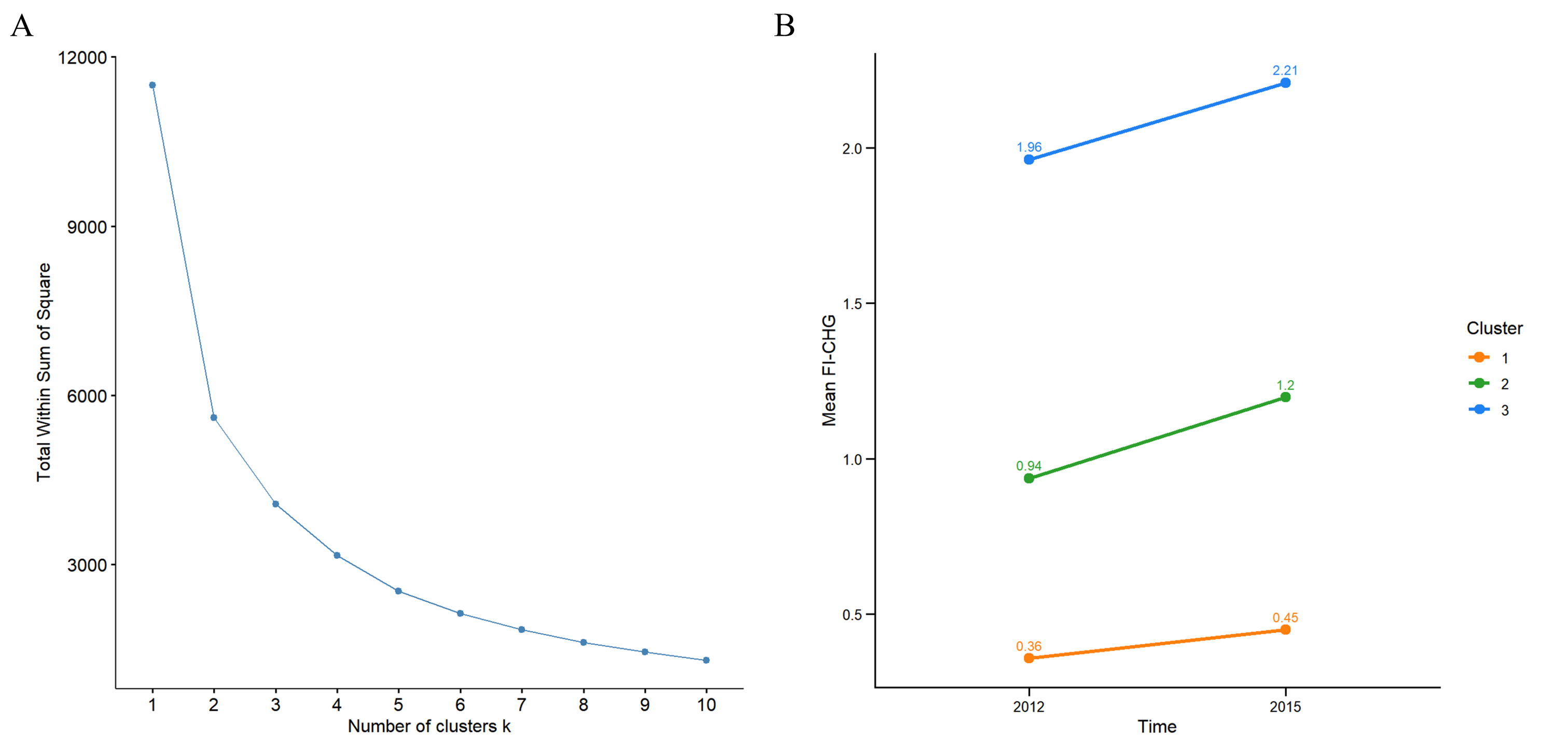
**

A.Three clusters were found using the K-means method; B. The change of FI-CHG from 2012 to 2015.

**Figure S2.** Comparison of the predictive value of FI, CHG and FI-CHG for CMM risk.


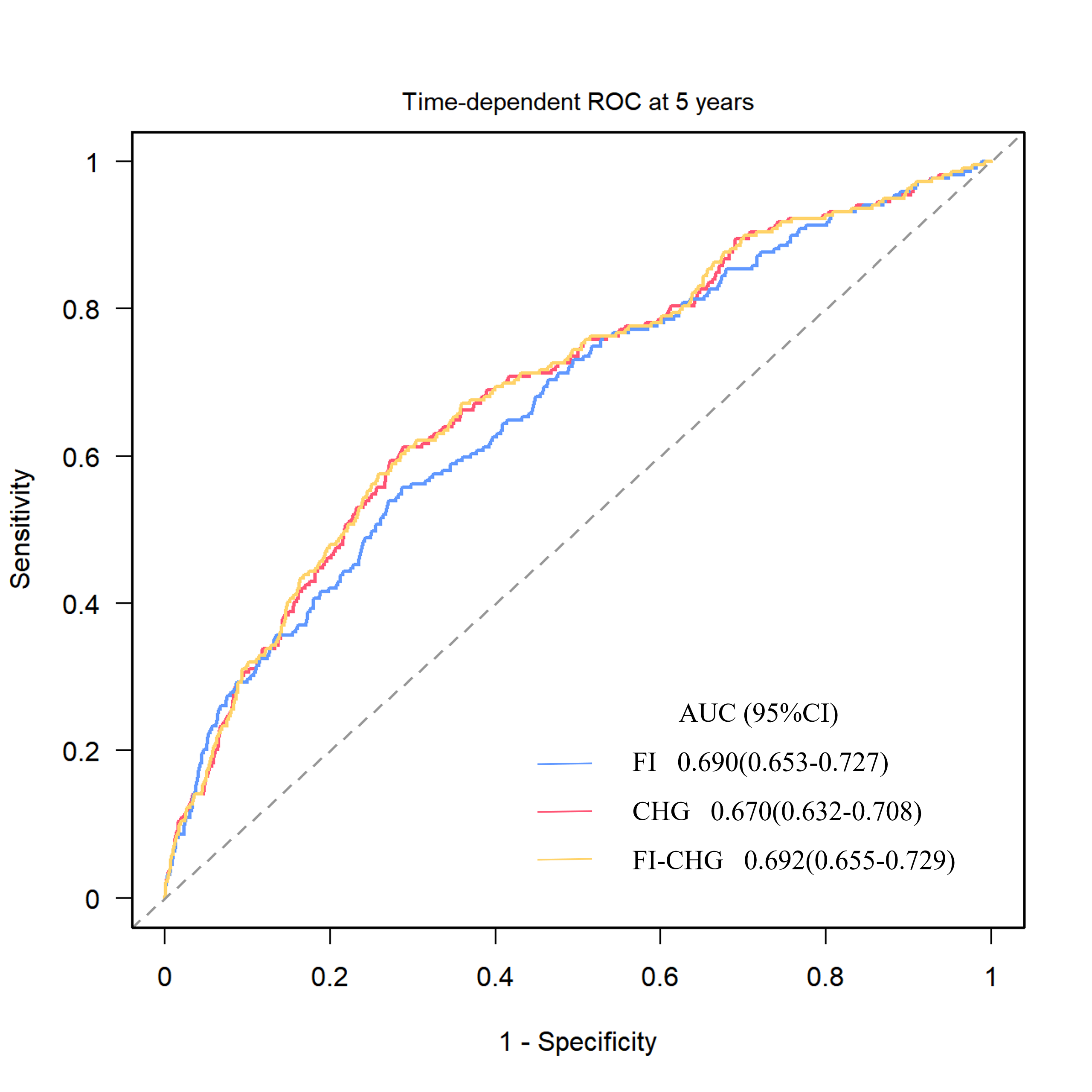


Abbreviations: FI, frailty index; CHG, Cholesterol, High-Density Lipoprotein, Glucose index; cumFI-CHG, cumulative FI-CHG; CMM, cardiometabolic multimorbidity; AUC, area under the curve; CI, confidence interval.

**Figure S3.** Age-associated risk of baseline FI-CHG for CMM incidence.


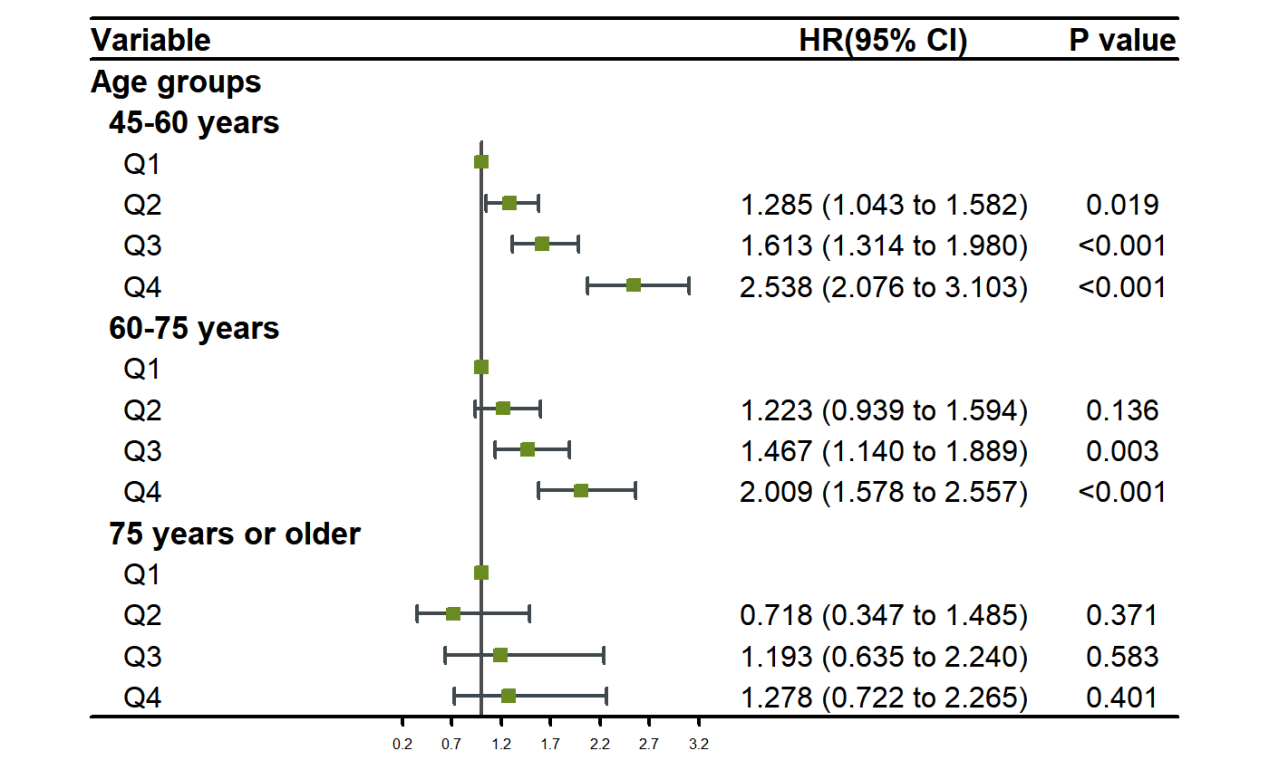


Model: Age, gender, location, education, marital, smoking, drinking, dyslipidemia, CRP, UA, and HbA1c were adjusted.

Abbreviations: FI, frailty index; CHG, Cholesterol, High-Density Lipoprotein, Glucose index; cumFI-CHG, cumulative FI-CHG; CMM, cardiometabolic multimorbidity

**Figure S4.** Age-associated risk of cumFI-CHG for CMM incidence.


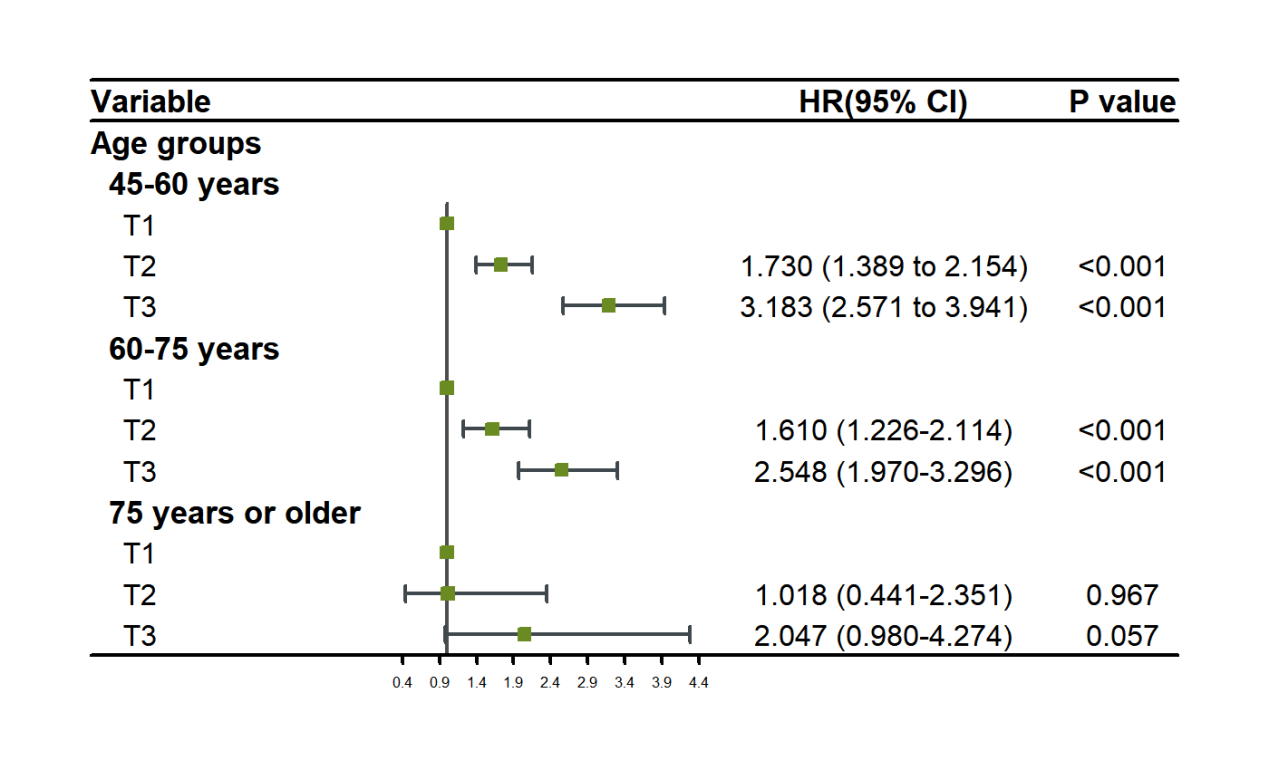


Model: Age, gender, location, education, marital, smoking, drinking, dyslipidemia, CRP, UA, and HbA1c were adjusted.

Abbreviations: FI, frailty index; CHG, Cholesterol, High-Density Lipoprotein, Glucose index; cumFI-CHG, cumulative FI-CHG; CMM, cardiometabolic multimorbidity.
